# Mechanistic Study of the Carbonylation of Aziridines to β‐Lactams: Alkylation, Solvent and Molecular NaBr and MgO Clusters Catalysts Effects

**DOI:** 10.1002/jcc.70061

**Published:** 2025-02-13

**Authors:** Abir Jendoubi, Mohamed Oussama Zouaghi, Youssef Arfaoui, Frédéric Guégan, Muneerah Mogren Al‐Mogren, Majdi Hochlaf

**Affiliations:** ^1^ Laboratoire de Caractérisations, Applications et Modélisation de Matériaux (LR18ES08), Faculté de Sciences de Tunis Université de Tunis El Manar Tunis Tunisia; ^2^ Université Gustave Eiffel, COSYS/IMSE Champs Sur Marne France; ^3^ IC2MP UMR 7285, Université de Poitiers—CNRS Poitiers France; ^4^ Department of Chemistry, College of Sciences King Saud University Riyadh Saudi Arabia

**Keywords:** carbonylation, catalysts, DFT, Mecanism, solvent

## Abstract

Using first principles methodology, we show that molecular NaBr and MgO nanoclusters are efficient ecofriendly and transition metal free catalysts for the carbonylation of aziridines to β‐lactams. Multi‐step mechanisms are proposed, where the activation energies of the rate‐determining step are strongly lowered compared to gas phase reaction, exhibiting, however, a unique step. Also, these reactions are viewed to be favored thermodynamically in the presence of these catalysts and also in solvents, in particular in methanol. We suggest thus the use of NaBr and MgO nanoclusters for these reactions. Besides, the present findings allow to explain the experimentally observed regioselectivity of carbonylation of aziridines reactions, where CO is added on the more substituted C of aziridine three‐membered ring. In sum, our work should motivate the use of such ecofriendly catalysts for carbonylation reactions without using transition metal catalysts.

## Graphical abstract

Using first principles methodologies, we treated the effects of non‐transition metal ecofriendly catalysts and solvents on the CO addition to aziridines. Computations show that both the thermodynamical and the kinetics are improved by sing such catalysts.

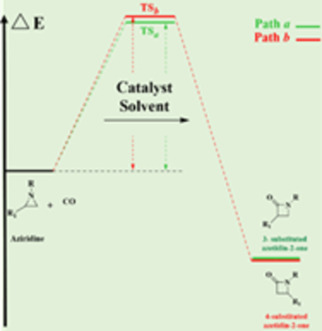

## 1 Introduction

Azetidin‐2‐ones, also known as β‐lactams, are cyclic amides consisting of a four‐membered ring. They are chemically categorized as 2‐azacyclobutanones. They derive their name from the attachment of the nitrogen atom to the β‐carbon relative to the carbonyl group. This structural motif is a recurring feature in numerous broad‐spectrum β‐lactam antibiotics and exhibits a wide range of other biological activities, including antibacterial, antitubercular, antifungal, anti‐inflammatory, antioxidant, anticonvulsant, antidepressant, and activity against Parkinson's disease [1, 2, 3, 4, 5, 6]. For more than a century, the distinctive structure and therapeutic properties of β‐lactam antibiotics have continuously captivated the interest of synthetic chemists due to the diverse synthetic challenges they present. Indeed, the significance of β‐lactams greatly increased following Alexander Fleming's discovery of penicillin in 1928 [7], which contains such four‐membered amide ring as confirmed by x‐ray crystallography. In particular, this azetidin‐2‐one ring was identified as the pivotal structural motif responsible for the antibiotic activity, with penicillin and cephalosporins being the primary naturally occurring examples of β‐lactam antibiotics until around 1970 [8].

**SCHEME 1:**
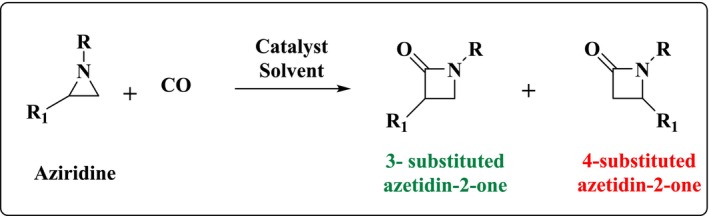
Chemical fixation of CO with aziridines to form substituted azetidin‐2‐ones.

Back to 1907 [9], the first synthesis of an azetidin‐2‐one was proposed. Since then, various procedures were suggested to produce (substituted)‐four‐membered β‐lactam ring [10, 11, 12]. In particular, aziridines carbonylation was proposed as a powerful tool for the formation of functionalized four ring β‐lactams (Scheme 1). Efficient syntheses were published as can be seen in References [13, 14, 15, 16, 17, 18, 19, 20, 21, 22, 23, 24, 25, 26, 27, 28, 29, 30]. Also, these works showed that these reactions are facilitated by the use of organometallic catalysts such as cobalt tetracarbonyl anion, rhodium‐complexed dendrimers, rhodium(I) dicarbonyl chloride dimer, tetrakis (triphenylphosphine) palladium (0), … [30] Specifically, they pointed out that the three‐membered ring of aziridines has strong strain that makes these compounds adjusted to opening and thus favoring the expansion of this ring via insertion of carbon monoxide. These experimental works were complemented by few theoretical investigations elucidating the role of the organometallic catalysts and the mechanisms and the enantioselectivity of these reactions [31, 32]. Indeed, the use of a catalyst for the coupling reaction of carbon monoxide with aziridines significantly enhances its reactivity and selectivity, enabling the efficient synthesis of valuable compounds such as poly‐β‐peptoids through lowered activation barriers and stabilized reaction intermediates [33].

Various transition metals, including rhodium, palladium, nickel, iron, and cobalt, have been employed to catalyze these reactions, each offering distinct advantages and limitations. Rhodium and palladium catalyzed processes have demonstrated high regio‐ and stereospecificity, though substrate scope remains restricted [31]. Cobalt‐based carbonylation, on the other hand, presents a promising approach with fewer substrate limitations, albeit with challenges in regio‐isomer formation [17]. Additionally, rhodium‐complexed dendrimers supported on resins have shown potential as efficient, recyclable catalysts for these transformations [28].

Although there are numerous experimental works dealing with the coupling reactions of carbon monoxide with aziridines, theoretical investigations are limited. Such theoretical studies could shed light on the underlying mechanisms and the site specificities of such reactions. In the present work, we perform in‐depth first principles treatments of the reactions between substituted aziridines and CO forming azetidine‐2‐one derivatives. We considered these reactions in gas phase, in the presence of solvents and catalysts. In quest of transition metal free catalysts, we focused here on molecular NaBr and MgO clusters as catalysts. The choice of NaBr is motivated by its role in green chemistry and being ecofriendly. For MgO clusters, we were inspired by previous works which pointed out the use of MgO catalytic surfaces for reactions between epoxide derivatives and aziridines with CO_2_ [34]. In addition, Chintareddy and Kantam [35] showed that MgO nanoparticles, with their relatively large surface area and active Mg^2+^ ions, can also significantly enhance the efficiency of the reactions between aziridines and CO by facilitating key reactions such as cycloadditions and ring openings. Furthermore, a hierarchical MgO microsphere catalyst, with its specific surface area and acidic/basic site concentrations, has demonstrated excellent adsorption properties, facilitating thus the tandem fixation of CO_2_ or CO into oxazolidinone under solvent‐free conditions and the subsequent addition of CO into aziridine [36].

## 2 Computational Details

Theoretical studies in previous works [31, 32] often employed mixed basis sets for different elements to optimize computational efficiency and accuracy. However, in our study, we utilize a uniform basis set across all elements in conjunction with density functional theory (DFT) to ensure consistency and simplify the interpretation of results. This approach is expected to provide more reliable and comparable data for the system under investigation [37].

We performed computations using DFT based approaches as implemented in GAUSSIAN 16 suite of programs (version C.01) [38]. The molecular structures were optimized using DFT, where we used various exchange‐correlation functionals: GGA PBE, hybrid B3LYP [39, 40], meta‐hybrid M06‐2X [41], and range separated ωB97X‐D [42, 43]. The atoms were described using the 6‐31G (d), 6‐31+G (d), 6‐311++G (d,p) and 6‐311++G (2d,2p) basis sets. We also performed explicitly correlated coupled clusters singles and doubles with perturbative treatment of triple excitations (CCSD(T)‐F12 [44, 45, 46]) single point computations as implemented in MOLPRO [47], where the atoms were described by the aug‐cc‐pVDZ basis set [48, 49] and the corresponding MOLPRO's default choices for the resolution of identity and density fitting basis sets [50]. As well established in the literature, the CCSD(T)‐F12 method [44] accelerates the convergence of basis sets, enabling results comparable to quadruple‐zeta quality in conjunction with the standard version (CCSD(T)) with the smaller aug‐cc‐pVDZ basis. Therefore, it can be used for benchmarking quantum chemistry techniques as done for instance in References [51, 52, 53, 54]. Geometries were here taken from optimizations at the B3LYP/6‐31+G(d) level of theory, revealed by our preliminary analyses as an appropriate level of theory (see hereafter).

Afterwards, we calculated the vibrational harmonic frequencies using the procedure as implemented in GAUSSIAN 16. These frequency calculations were conducted, to characterize the molecular structures, whether these structures represent minima (with no imaginary frequencies) or transition states (exhibiting only one imaginary frequency). Additional analyses were carried out using intrinsic reaction coordinate (IRC) [55, 56, 57] computations to confirm that the transition‐state structures indeed connect the expected reagents and products. Furthermore, we carried out in‐depth analysis of the reaction force constants for each mechanism in order to get insights onto their synchronicity [58, 59].

To account for solvent effects, single‐point self‐consistent reaction field (SCRF) calculations were done using the conductor‐like polarizable continuum model (CPCM) [60, 61, 62].

## 3 Results and Discussion

We started our theoretical investigations by systematic studies on the atomic basis sets and DFTs in order to select a method of choice for treating the coupling reactions between aziridines and CO. First, four functionals (PBE, B3LYP [39, 40], M06‐2X [41], and ωB97X‐D [42, 43]) in conjunction with the 6‐31+G(d) basis set were evaluated, where the data at the CCSD(T)‐F12/aug‐cc‐pVDZ were used as reference. As shown in Table 1, B3LYP offers here the closest reproduction of the reference energetics, both considering the activation and reaction energies, whereas a small difference between Δ*E*_r_ values computed using B3LYP and CCSD(T)‐F12 is noticed. We can note a non‐negligible underestimation of energies at the GGA level, which is quite expected as discussed in Reference [63]; conversely, hybrids tend to overestimate the activation energy, M06‐2X and ωB97X‐D rather largely overshooting the reference CCSD(T)‐F12 value. In addition, the optimized structures at the B3LYP/6‐31+G(d) and CCSD(T)‐F12/aug‐cc‐pVDZ levels of the reactants and the products of the reactions forming 3‐methyl‐azetidin‐2‐one and 4‐methyl‐azetidin‐2‐one are very close (cf. Figure S1). As a result, in the following we selected B3LYP as our reference DFT method.

**TABLE 1:** Computed reaction energies (Δ*E*_r_, kcal mol^−1^) and activation energies (*E*_a_, kcal mol^−1^) for the gas phase synthesis of 3‐methyl‐azetidi‐2‐one and 4‐methyl‐azetidi‐2‐one derivatives by the reaction of a substituted aziridine with CO using different DFT functionals in conjunction with the 6‐31+G (d) basis set and the CCSD(T)‐F12/aug‐cc‐pVDZ method.

Compound	CCSD(T)‐F12		PBE		B3LYP		M06‐2X		ωB97X‐D	
	*E*_a_	Δ*E*_r_	*E*_a_	Δ*E*_r_	*E*_a_	Δ*E*_r_	*E*_a_	Δ*E*_r_	*E*_a_	Δ*E*_r_
3‐Methyl‐azetidin‐2‐one	51.2^a^	−34.0^a^	41.0	−40.6	58.0	−34.0	68.3	−28.5	63.2	−36.3
	—	−27.8^b^								
4‐Methyl‐azetidin‐2‐one	59.5^a^	−29.7^a^	‐^c^	−41.9	59.2	−35.4	68.1	−30.0	63.3	−37.8
		−29.4^b^								

^a^ Single point computations at the B3LYP/6‐31+G(d) optimized geometries.

^b^ After full optimization.

^c^ Convergence problems.

Next, four different basis sets (6‐31G(d), 6‐31+G(d), 6‐311++G(d,p), and 6‐311++G(2d,2p)) were employed to describe the atoms and identify a sufficiently large basis set for describing the reactants, the intermediates (if any), the transition states, and the products. The selection process was based on comparing the activation energy values for the synthesis of β‐lactam derivative via the coupling of aziridine with CO. As shown in Table 2, the 6‐31+G(d) basis set displayed the lowest *E*_a_ value and the closest data to those obtained with CCSD(T)‐F12/aug‐cc‐pVDZ. This indicates the best performance of 6‐31+G(d) among the tested basis sets. Hereafter, this basis will be used.

**TABLE 2:** Computed reaction energies (Δ*E*_r_, kcal mol^−1^) and activation energies (*E*_a_, kcal mol^−1^) for the gas phase synthesis of a 3‐methyl‐azetidine‐2‐one derivative by the reaction of a substituted aziridine with CO using the B3LYP DFT in conjunction with different basis sets.

Product	3‐Methyl‐azetidin‐2‐one	
Basis set	*E*_a_	Δ*E*_r_
6‐31G (d)	60.0	−32.4
6‐31+G (d)	58.0	−34.0
6‐311++G (d,p)	58.2	−31.6
6‐311++G (2d,2p)	59.2	−29.2
CCSD(T)‐F12/aug‐cc‐pVDZ	51.2	−34.0

*Note:* We also give the CCSD(T)‐F12/aug‐cc‐pVDZ single point computations data as done at the B3LYP/ 6‐31+G(d) optimized geometries.

### 3.1 Non‐catalyzed Cycloaddition Reaction With CO

**FIGURE 1:**
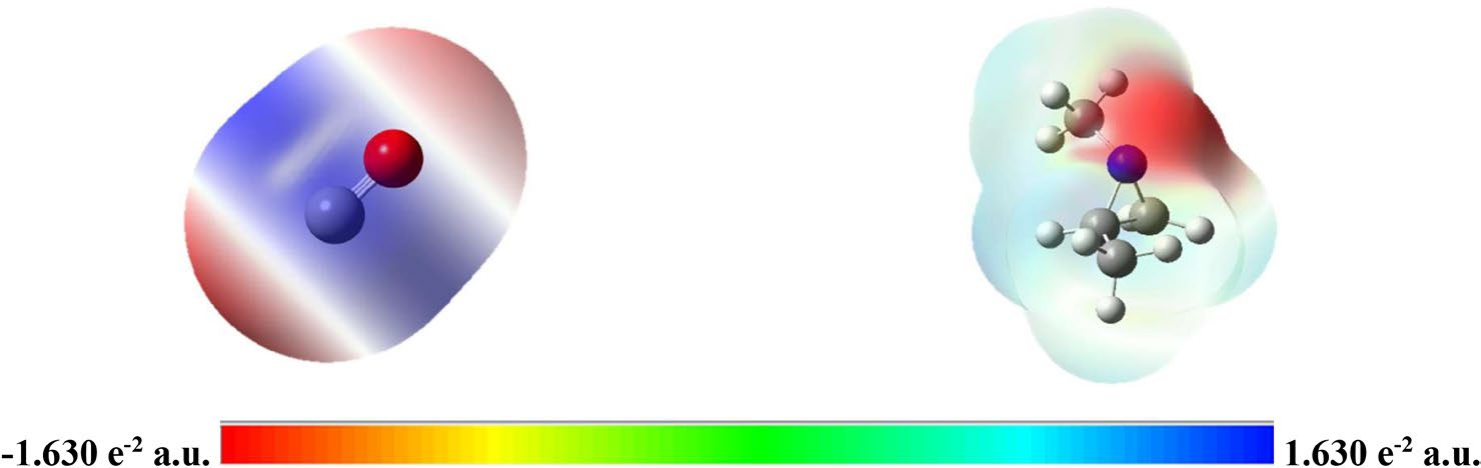
B3LYP/6‐31+G(d) optimized structures and computed 3D electrostatic potential surface maps (3D MEPS, isovalue of 0.0004 a.u) of CO (left) and aziridine (right) in the gas phase.

**SCHEME 2:**
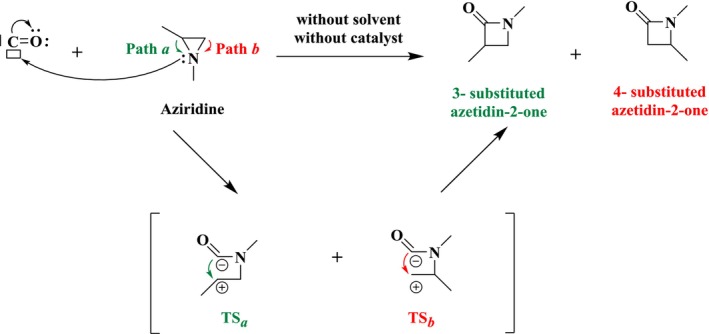
Proposed reaction mechanism for non‐catalyzed cycloaddition reaction of CO with aziridine.

To assess the role of catalysts, the coupling reaction between CO and aziridine to produce β‐lactam was studied without the presence of a catalyst. The transition state for this reaction was identified at the B3LYP/6‐31+G(d) level in the gas phase. Initially, the molecular structures were optimized at the B3LYP/6‐31+G(d) level, as depicted in Figure 1. This figure also shows the 3D electrostatic potential surface maps (3D MESP) of CO and aziridine. These 3D MESPs illustrate the electronic properties of the molecules by highlighting regions of positive and negative electrostatic potential, which help in understanding noncovalent interactions by identifying reactive sites [64, 65]. As shown in Figure 1, the carbon atom of CO has a relatively large positive potential, indicating it as the most electrophilic site, whereas the nitrogen atom of aziridine shows a significant negative potential. Consequently, the carbon atom in CO is prone to nucleophilic attack from the lone pair of the aziridine nitrogen. Accordingly, the carbonylation of aziridines, leading to the synthesis of azetidin‐2‐ones, can proceed in a single step without a catalyst. This reaction mechanism is illustrated in Scheme 2.

**FIGURE 2:**
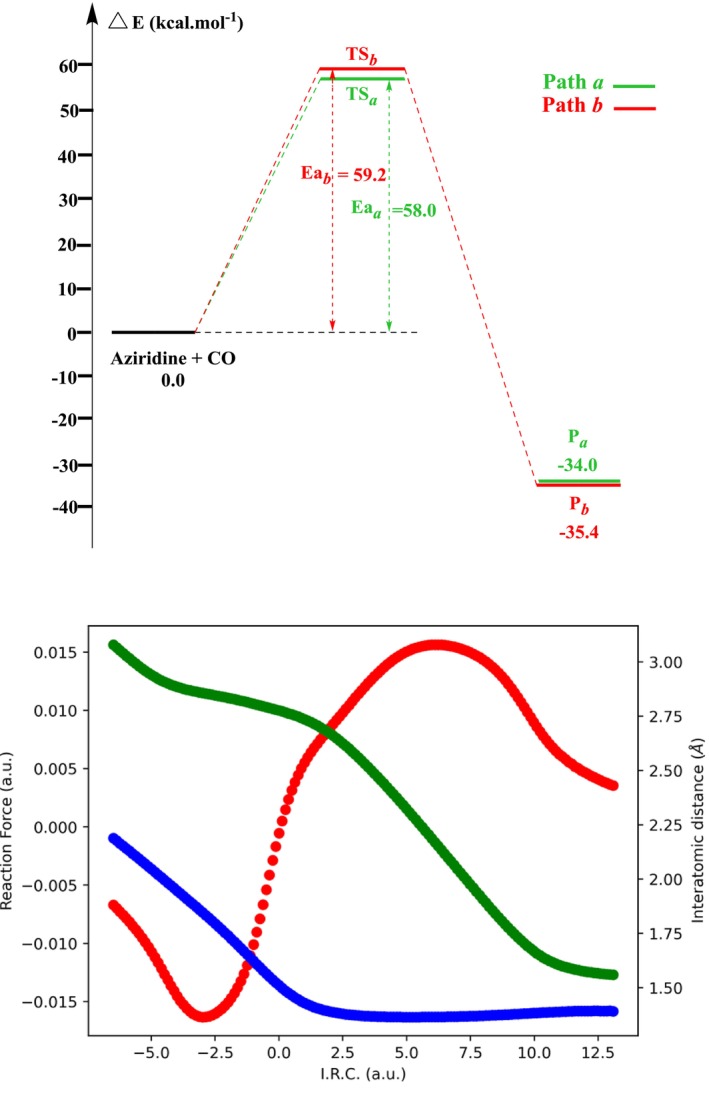
Top: Reaction energy profiles for noncatalyzed cycloaddition reaction of CO with aziridine calculated at the B3LYP/6‐31+G(d) level in the gas phase. Bottom: Evolution of the reaction force (red, in atomic units), C–N (blue, Å) and C–C (green, Å) bond lengths along the IRC in the vacuum.

Scheme 2 shows that the carbonylation reactions of aziridines initiate by coordinating a CO molecule to the nitrogen atom of the aziridine, followed by a nucleophilic interaction between the carbon atom of the coordinated CO and the carbon atom of aziridine. Therefore, two possible pathways, Path *a* and Path *b*, were considered, given that aziridine comprises two non‐equivalent carbon atoms, denoted as C*a* (the more substituted carbon) and C*b* (the less substituted carbon). In Path *a*, the nucleophilic attack starts on C*a*, while in Path *b*, it begins from C*b*. Each pathway has a single transition state, TS_*a*_ or TS_*b*_, which connects the cleavage of the C*a*–N or C*b*–N bonds and the formation of the C*a*–O or C*b*–O bonds, respectively. Each pathway leads to a distinct β‐lactam product: Path *a* generates a 3‐substituted azetidine‐2‐one (P*a*), while Path *b* produces a 4‐substituted azetidine‐2‐one (P*b*). The corresponding energy profiles are depicted in Figure 2. Overall, both reactions are exothermic, with energy values of −34.0 and −35.4 kcal mol^−1^ for Path *a* and Path *b*, respectively. Considering the calculated energy barriers, Path *a* (58.0 kcal mol^−1^) is slightly kinetically favored over Path *b* (59.2 kcal mol^−1^). The difference in barrier heights between both pathways can be rationalized using the tentative Lewis formulas depicted in Scheme 2: Path *b* is associated with the formation of a formal primary carbocation, while Path *a* displays a secondary carbocation, hence is more stabilized.

Figure 2 shows also the evolutions of the reaction force, the C–N and C–C bond lengths along the IRC. As can be seen from this figure, in the vicinity of the transition states TS_*a*_, the C–N bond is already significantly formed, with a C–N distance nearing 1.50 Å. Conversely, the C–C bond distance is still rather large (above 2.50 Å). The TS region is thus associated with a switch from the formation of the C–N bond to the formation of the C–C bond, which can be associated with the change of slope of the reaction force profile after the TS. The reaction in vacuum can thus be seen as being concerted but already quite asynchronous. Anyway, these potential barriers are quite large. As discussed in References [10, 30, 32]. introducing appropriate catalysts and/or solvents is advisable to reduce the energy barriers of the reaction.

### 3.2 Solvent Effects

In the cyclization step, the choice of solvent plays a crucial role in determining the reaction outcome, as evidenced by both experimental and computational studies. Organic solvents such as Et_3_N in MeOH showed to lead to a mixture of products due to competing reaction pathways, while changing to an aqueous solution can steer the reaction toward a single desired β‐lactam product [66]. This highlights the importance of organic solvents in influencing the direction and efficiency of chemical reactions.

We investigated the cycloaddition reaction of CO with aziridine while considering the influence of different solvents via CPCM implicit solvation model. The corresponding reaction energetics are given in Table 3, which shows that 3‐methyl‐azetidin‐2‐one is both thermodynamically and kinetically favored over 4‐methyl‐azetidin‐2‐one, as observed in gas phase. Remarkably, the implementation of the implicit solvation model reduces Δ_r_ *E* and *E*_a_ values for both reaction pathways, especially in methanol, where Δ_r_ *E* decreases from −34.0 to −38.6 kcal mol^−1^ (Path *a*) and *E*_a_ is lowered from 58.0 to 47.8 kcal mol^−1^. For Path *b*, we observe similar energy amount of reduction (in absolute values). A clear linear relationship between activation energies and the inverse of the dielectric constants of solvents can further be evidenced, which is consistent with the presence of formal charges in the transition state as proposed before. However, since these charges are “buried” within the molecules and less accessible to the external solvent, the effect is rather limited. In fact, extrapolation of the previous linear model to infinite polarity only results in activation barriers of 47.5 and 50.0 kcal mol^−1^ for Paths *a* and *b*, respectively—values that are still too high to allow this reaction to occur in standard conditions. The joint use of a polar solvent and of a catalyst thus seems required here.

**TABLE 3:** Computed reaction energies (Δ*E*_r_, kcal mol^−1^) and activation energies (*E*_a_, kcal mol^−1^) for the synthesis of 3‐methyl‐azetidin‐2‐one and 4‐methyl‐azetidin‐2‐one products by the reaction of a methyl aziridine with CO at the B3LYP/6‐31+G(d) level in different solvents (CPCM).

Solvent	*ε*_r_	3‐Methyl‐azetidin‐2‐one		4‐Methyl‐azetidin‐2‐one	
		*E*_a_	Δ_r_ *E*	*E*_a_	Δ_r_ *E*
Methanol	32.7	47.8	−38.6	50.2	−40.2
Dichloro‐methane	8.9	48.9	−41.2	51.3	−39.7
Diethyl ether	4.3	50.6	−37.6	52.7	−39.0
Toluene	2.4	52.8	−36.7	54.6	−38.0

*Note:* ε_r_ is the dielectric constant of the solvent.

### 3.3 Aziridine Substituent Effects

Alternatively, activation of the substrate could be conceived by derivatization. Various derivatives of 3‐ and 4‐azetidin‐2‐ones, each di‐substituted with different electronic effects (both mesomeric and inductive), were investigated to assess their impact on the reaction energy (Δ_r_ *E*) and the activation energy (*E*_a_) values. Their thermodynamic and kinetic profiles were then analyzed and detailed in Table 4. All computations were carried out at the B3LYP/6‐31+G(d) level of theory in methanol as implicit solvent.

**TABLE 4:** Computed reaction energies (Δ*E*_r_, kcal mol^−1^) and activation energies (*E*_a_, kcal mol^−1^) for the synthesis of 3‐substituted azetidine‐2‐one and 4‐substituted azetidin‐2‐one derivatives by the reaction of a substituted aziridine with CO calculated at B3LYP/6‐31+G(d)/CPCM (methanol) level.

R, R_1_	3‐Substituted azetidin‐2‐one		4‐Substituted azetidin‐2‐one	
	Δ*E*_r_	*E*_a_	Δ*E*_r_	*E*_a_
H, CH_3_	−35.3	48.6	−37.0	51.8
CH_3_, CH_3_	−38.6	47.8	−40.2	50.2
OCH_3_, CH_3_	−29.6	50.9	−31.4	52.8
COCH_3_, CH_3_	−33.7	48.7	−34.1	56.7
CH_3_, CH_2_Ph	−38.3	47.7	−39.2	50.5
CH_3_, CH_2_Cl	−37.8	48.1	−38.6	52.5

*Note:* Cf. Scheme 1 for the definition of R and R_1_.

Table 4 gives the reaction energies (Δ*E*_r_) and activation energies (*E*_a_) for the synthesis of 3‐ substituted azetidine‐2‐one and 4‐ substituted azetidin‐2‐one derivatives by the reaction of a substituted aziridine with CO as computed at the B3LYP/ 6‐31+G(d)/CPCM (methanol) level. This table shows that the substituents on the aziridine ring significantly influence both the reaction and activation energies for the formation of 3‐substituted and 4‐substituted azetidin‐2‐one derivatives. Substituents with electron‐donating groups, such as methyl (CH_3_), result in lower activation energies and more negative reaction energies, suggesting that these substituents stabilize the transition state and the product, thereby facilitating the reaction. Conversely, substituents with electron‐withdrawing properties, such as the methoxy group (OCH_3_), increase the activation energy, as seen in the higher *E*_a_ values (50.9 kcal mol^−1^ for 3‐substituted and 52.8 kcal mol^−1^ for 4‐substituted azetidin‐2‐one). This indicates that these substituents destabilize the transition state, making the reaction less favorable. The reaction energies are also less negative for these groups, documenting less thermodynamically favorable process.

In the following, we chose to further investigate the di‐methylated aziridine compound (i.e., R = CH_3_ and R_1_ = CH_3_) forming thus 3‐ and 4‐methyl‐azetidine‐2‐ones, as they offer promising characteristics.

### 3.4 Carbonylation Reaction Catalyzed by NaBr

In 2008, Mu et al. [67] investigated the LiI‐catalyzed coupling of carbon dioxide and aziridine reaction using DFT and MP2 calculations. This reaction leads to the formation of oxazolidinones. According to their proposed mechanism, the overall reaction occurs in two‐steps: the formation of a trimolecular complex involving aziridine, CO_2_, and LiI, followed by ring opening and subsequent closure to yield the oxazolidinone. They also showed that various factors, including the electronic and steric effects of the aziridine functionality, the Lewis basicity, and steric volume of the nucleophile and/or metal catalyst, play significant roles in determining the preferred isomer. Here, we incorporate NaBr as a catalyst in the gas phase carbonylation of aziridine. As LiI, NaBr is a green ecofriendly catalyst, which is in contrast to transition metal‐based organometallic ones, as those used previously [17, 67, 68].

**SCHEME 3:**
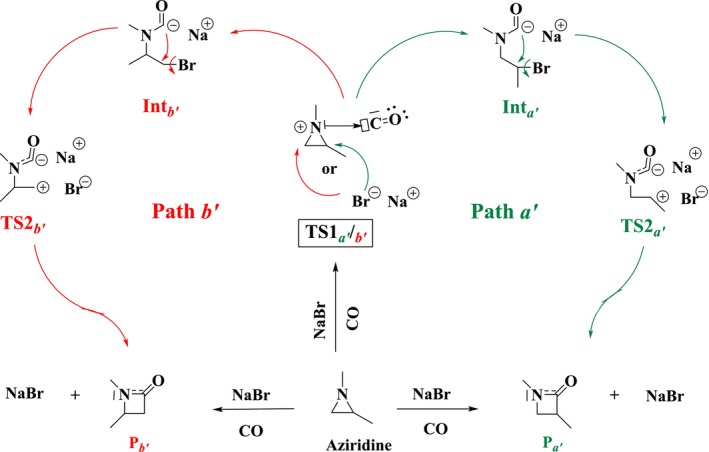
Proposed reaction mechanism for CO and aziridine coupling reaction to oxazolidinones catalyzed by NaBr alone.

**FIGURE 3:**
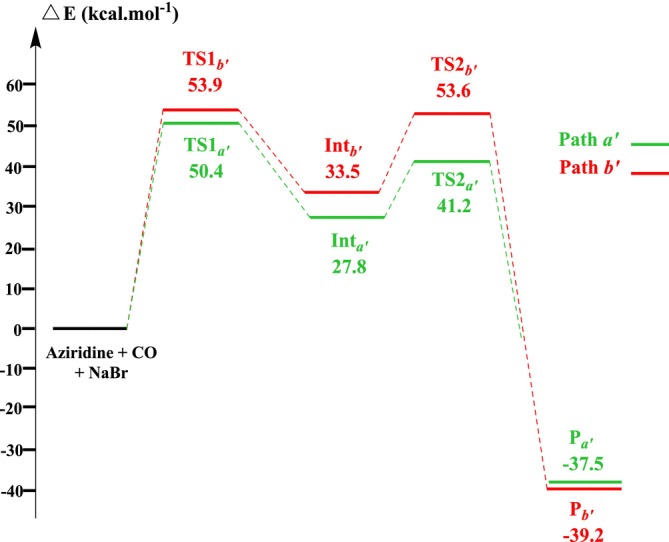
NaBr‐catalyzed reaction energy profiles covering the intermediates and transition states calculated at the B3LYP/6‐31+G(d) level in gas phase.

In Scheme 3, we present a mechanism for this reaction, where we were inspired by the mechanisms proposed by Mu et al. [67] and Jendoubi et al. [69]. The corresponding computed energy diagram is given in Figure 3. Two pathways are considered here too, related to the nature of the cleaved N‐C bond. In the NaBr‐catalyzed case, the carbon monoxide behaves like a Lewis acid by coordinating with the nitrogen atom of the aziridine (electrophilic assistance). This interaction forms a complex between CO and aziridine (Scheme 3) which is activated toward a nucleophilic substitution reaction occurring at the C*a′/b'* atom of aziridine and involving a bromide anion. This substitution leads to the formation of an adduct that can then undergo a second nucleophilic substitution from the acylide, resulting in the expected 3‐ methyl‐azetidin‐2‐one or 4‐methyl‐azetidin‐2‐one and regeneration of NaBr.

**SCHEME 4:**
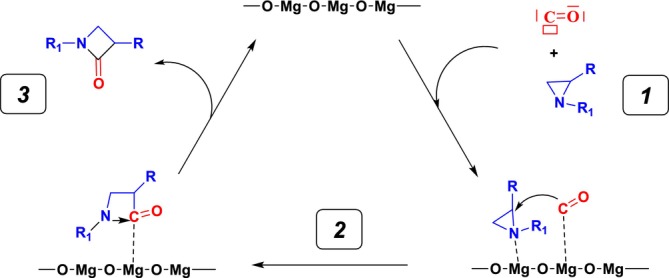
Mechanism of the MgO‐catalyzed reaction of CO and aziridine: ***1*** corresponds to adsorption of CO and aziridine, ***2*** is for the aziridine ring opening and ***3*** leads to formation of the azetidin‐2‐one.

It may here be noted that the overall reactions proceed with the same stereochemistry as the gas‐phase one. Indeed, although the reaction is sequential (two‐steps) and no longer concerted‐asynchronous, the fact that it relies on second‐order nucleophilic substitution steps ensures a full retention of stereochemistry.

Figure 3 shows that the first step is the rate‐determining one in both cases, with an activation energy for Path *b'* of 53.9 kcal mol^−1^, slightly higher than that for Path *a'* (50.4 kcal mol^−1^). This indicates a small kinetic preference for the formation of the 3‐substituted azetidine‐2‐one over 4‐substituted azetidine‐2‐one. Nevertheless, activation barriers are still too high here to make the reaction feasible in experimental conditions.

### 3.5 Cycloaddition Reaction Catalyzed by MgO Clusters

Initially, our focus was on identifying the most suitable theoretical method for optimizing the MgO clusters. Studies were thus performed on Mg_*n*_O_*n*_ (*n* = 8, 16) clusters miming the MgO (001) crystal surface (cf. Figures S2 and S3). This surface crystal was chosen due to its stability and prevalence, making it a representative model for investigating adsorption interactions and catalytic properties in various applications [13].

Overall, our calculations (see Supporting Information for more information) reveal the most appropriate model to reproduce the structure of the MgO solid is a bilayer Mg_16_O_16_ slab. Indeed, the smaller Mg_8_O_8_ slab is subject to deformations upon optimizations or interaction with CO molecule whereas the Mg_16_O_16_ slab structure remains close to that of MgO surface (cf. Figures S4 and S5 and Supporting Information for more details). These findings are determined at the PBE/6‐31+G(d) level of theory. Nevertheless, as seen earlier this approach is likely to underestimated activation barriers, and energies obtained with PBE are incomparable to those obtained using B3LYP. In the following, we thus opted for a composite method, computing energies at the B3LYP/6‐31+G(d) level on PBE/6‐31+G(d) structures.

**FIGURE 4:**
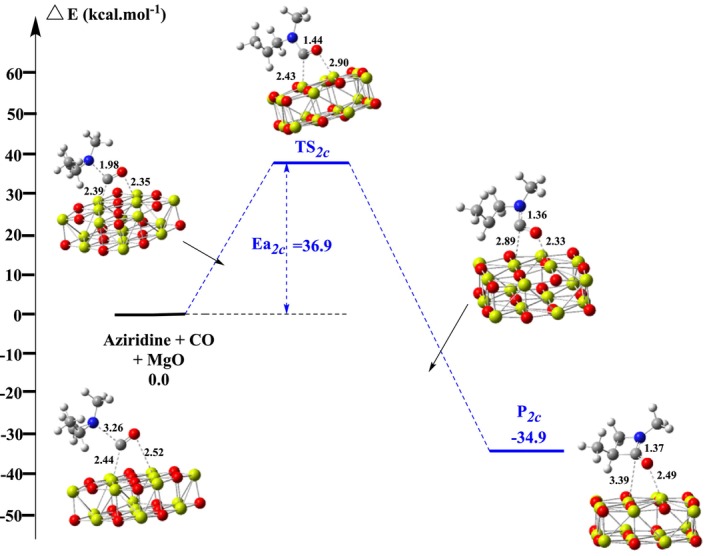
Energy diagram of azetidi‐2‐one synthesis catalyzed by bilayer Mg_16_O_16_ cluster surface on Mg in the middle of the surface. Distances are in Å.

**FIGURE 5:**
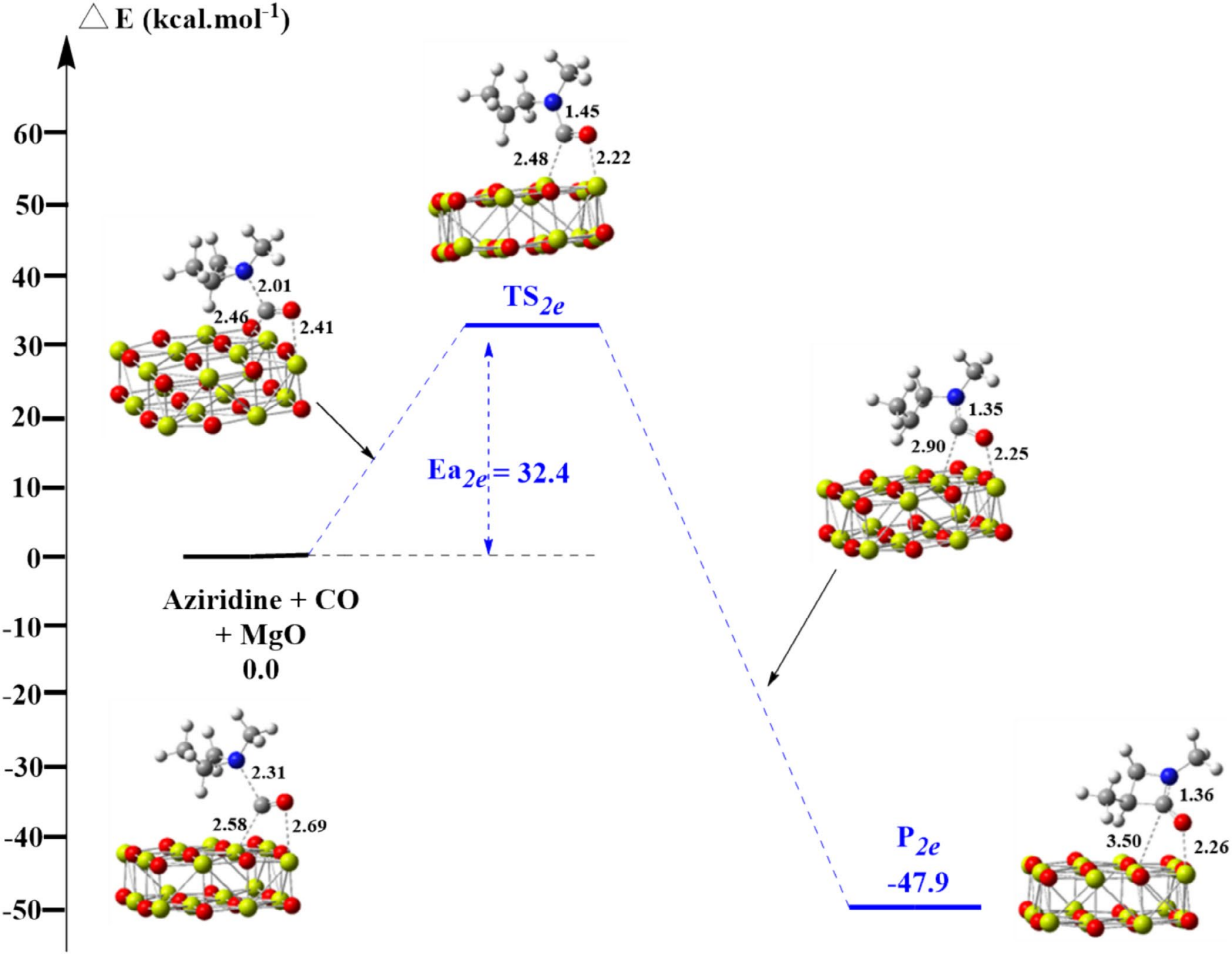
Energy diagram of azetidi‐2‐one synthesis catalyzed by bilayer Mg_16_O_16_ cluster surface on Mg at the surface end. Distances are in Å.

We then evaluated the kinetic and thermodynamic profile of the CO insertion in aziridine occurring at the surface of the Mg_16_O_16_ cluster surface at the B3LYP/6‐31+G(d)//PBE/ 6‐31+G(d) level. Here, only the formation of the 3‐azetidine‐2‐one product could be observed, but two reaction profiles can nevertheless be considered depending on the location of the reagents on the MgO model surface: (*i*) either CO coordination to a Mg atom in the middle of the surface or (*ii*) on a Mg atom at the surface edge (cf. Figures S5 and S6). Geometric parameters and RMSD values are summarized in Table S4. The corresponding energy diagrams for both types of adsorptions are shown in Figures 4 and 5, Scheme 4. As one may note, in both cases a significant lowering in the activation energy is seen, activation barriers now nearing 30 kcal mol^−1^. Interestingly these quantities are smaller than the ones derived for such reactions in the presence of Rh or Co‐based catalysts [31, 32]. These findings suggest MgO clusters could be an efficient catalyst for the target reaction, although the strong stabilization of the product on the surface of MgO (as indicated by the larger exergonicity) suggests that catalyst passivation or poisoning could be an issue here.

**FIGURE 6:**
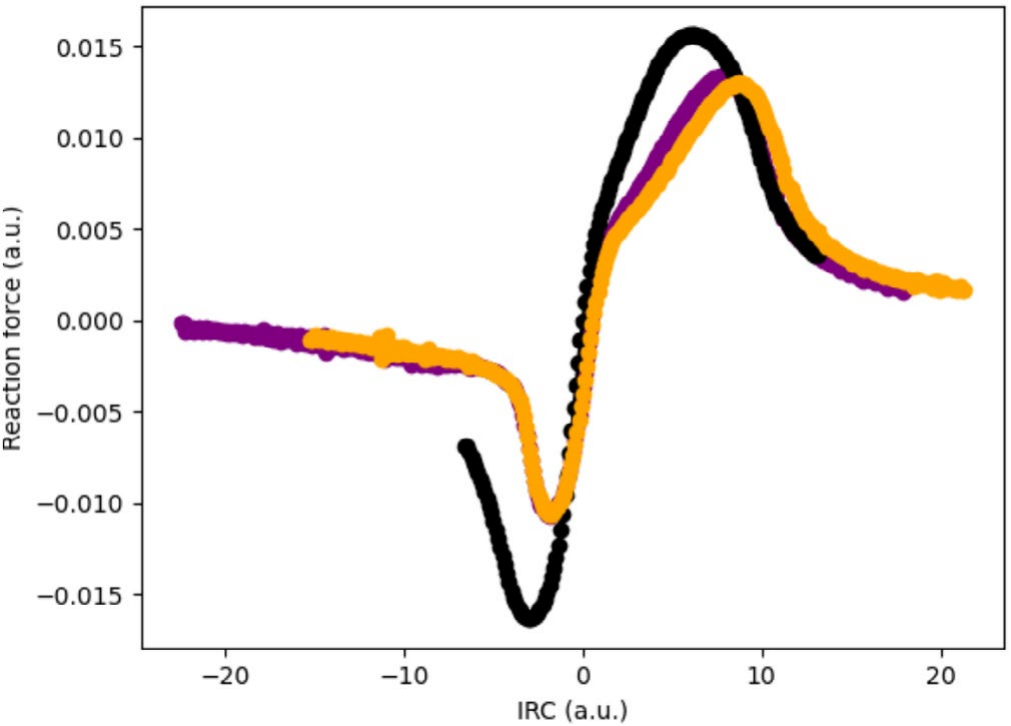
Reaction force profiles computed along the IRC for the reaction in vacuum (black), at the center of (i) the MgO slab (purple) or on (ii) the edge (orange). All values are in atomic units. See text for more details.

**FIGURE 7:**
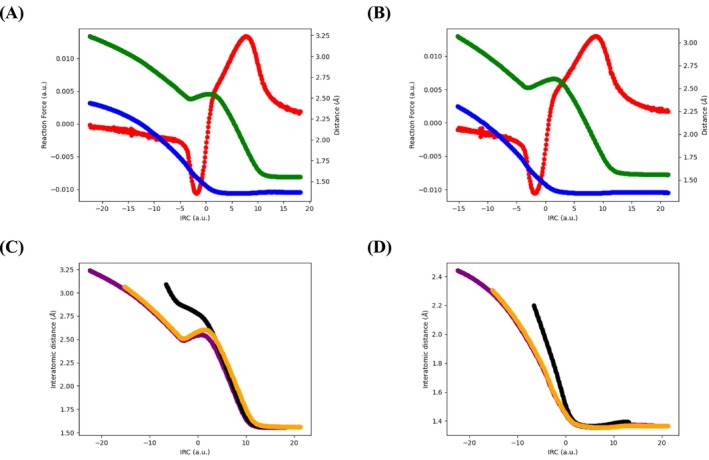
Top: Evolution of the reaction force (red, in atomic units), C–N (blue, Å) and C–C (green, Å) bond lengths along the IRC, for the reaction on the center (in (A)) or edge (in (B)) of the MgO slab. Bottom: Evolution of the C–C (in (C)) and C–N (in (D)) bond distances (in Å) along the IRC for the vacuum (black) and MgO‐catalyzed reactions (center, purple; edge, orange).

To get some insights on the effect of Mg_16_O_16_ catalyst, we analyzed the corresponding reaction force profiles and the evolution of C–C and C–N distances in the case of the catalyst free carbonylation of aziridines reaction and that in the presence of MgO cluster (Figure 6). As the reaction coordinates are largely comparable in the cases of the reactions performed in vacuum and in the presence of the catalyst (both at the center and edge of the slab), we may compare their reaction force profiles. The most striking difference between both profiles is found after the transition state: the presence of the catalyst is associated with a change of concavity, indicative of a larger asynchronicity of the reaction process. The difference between the (i) center and (ii) edge positions on the catalyst is at this stage not plain, both curves being rather similar as indicated in Figure 7. Since the reaction process principally involves the formation of two bonds (C–N and C–C), we further studied the evolution of these along the IRC.

Let us now consider the MgO‐catalyzed reactions. As one may note, as previously the C–N bond is already formed at the TS (distance slightly below 1.50 Å), while the C–C bond distance is still very large (around 2.50 Å) as indicated in Figure 7. While the evolution of the C–N distance is monotonous along the reaction profile, the C–C one presents a more surprising variation: it decreases from the reagent region to a point of the potential energy surface right before the TS, where it starts to increase slightly before dropping abruptly after the TS. These changes in variation directly connect to remarkable points on the reaction force profile (take‐off before TS and inflection point after it). We thus here evidence an effect induced by the catalyst. If we now compare the bond distances evolution, we note that the formation of the C–N bond occurs earlier on the reaction profiles in the catalyzed cases, while the C–C bond formation takes place at roughly the same point—notwithstanding the peculiar evolution around the transition state (Figure 7). Overall, the reaction is thus more asynchronous in the case of the MgO catalysis, as expected.

## 4 Conclusions

We investigated the carbonylation of aziridines using first principles methodology. After benchmarks, we showed that the reaction proceeds via a one‐step mechanism in gas phase where a regioselectivity is observed favoring the addition of CO on the most substituted C of the aziridine three‐membered ring. Inclusion of an implicit model of solvent reveals a moderate lowering of the activation energy supporting the use of polar solvent in conjunction with catalysts. We considered here ecofriendly and transition state‐metal free catalysts, namely molecular NaBr and MgO clusters. In the case of NaBr, a sequential mechanism is identified, involving successive second‐order nucleophilic substitution reaction enforcing stereochemical control on the product of reaction. In the case of MgO, a concerted mechanism is still evidenced, but presents a higher degree of asynchronicity, the catalyst helping here to decorrelate the elementary events occurring during the reaction (formations of C–C and C–N bonds). Overall, the capture of CO by aziridine in the presence of MgO nanoparticles and in polar solvents such as methanol may thus be efficient. We hope this work will steer further research from experimentalists.

## Conflicts of Interest

The authors declare no conflicts of interest.

## Supporting information


**Data S1.** Supporting Information.

## Data Availability

The data that supports the findings of this study are available in the Supporting Information of this article.
