# Supplementary material for: Mechanistic Study of the Carbonylation of Aziridines to β‐Lactams: Alkylation, Solvent and Molecular NaBr and MgO Clusters Catalysts Effects
Source: J Comput Chem. 2025 Feb 13;46(5):e70061. doi: 10.1002/jcc.70061 (PMC11823392; doi:10.1002/jcc.70061)
Supplement: Supplementary file 1 — Data S1. Supporting Information. [file JCC-46-0-s001.pdf]

Supplementary Information

Mechanistic Study of the Carbonylation of Aziridines to β-Lactams: Alkylation, Solvent and Molecular NaBr and MgO clusters catalysts effects

Abir Jendoubi,[a,d] Mohamed Oussama Zouaghi,[a] Youssef Arfaoui,[a,*] Frédéric Guégan,[b] Muneerah Mogren Al-Mogren,[c] Majdi Hochlaf [d,*]

[a] Laboratoire Applications, Caractérisations et Modélisation de Matériaux (LR18ES08), Faculté des Sciences de Tunis, Université de Tunis El Manar, Tunis, 2092, Tunisia
[b] IC2MP UMR 7285, Université de Poitiers – CNRS, 4, rue Michel Brunet TSA 51106–86073 Cedex 9 Poitiers, France
[c] Department of Chemistry, College of Sciences, King Saud University, PO Box 2455, Riyadh 11451, Saudi Arabia
[d] Université Gustave Eiffel, COSYS/IMSE, 5 Bd Descartes, 77454, Champs Sur Marne, France

Summary of Tables and Figures in Supplementary Information

Tables

Table S1: Theoretical geometrical parameters (in Å) of the Mg_8O_8 cluster (monolayer) with values of total energy (in Hartree), electronic gap (in eV) and RMSD (in Å) as calculated at the B3LYP/6-31G(d), B3LYP/6-31+G(d) and PBE/6-31+G(d) levels of theory. The numbering of the atoms is given in Figure S1. Distances are in Å.

Table S2: Theoretical geometric parameters (in Å) of the $Mg_{16}O_{16}$ bilayer surface calculated at PBE/ 6-31+G(d) level. The numbering of the atoms is given in Figure S2. Distances are in Å. RMSD is in Å.

Table S3: Geometric parameters (in Å) of the adsorption of CO on $Mg_{16}O_{16}$ bilayer cluster for both (*a*) CO and (*b*) OC configurations as optimized at the PBE / 6-31+G level. The numbering of the atoms is given in Figure S3. Distances are in Å.

Table S4: Geometric parameters (in Å) of the $Mg_{16}O_{16}$ bilayer surface calculated at PBE / 6-31+G (d) (*i*) on Mg at mid-surface (*ii*) on Mg at cluster edge. The numbering of the atoms is given in Figure S4. Distances are in Å.

Table S5: Optimized Coordinates for the synthesis of 3-methyl-azetidi-2-one derivative by the reaction of a substituted aziridine with CO using different DFT functionals.

Table S6: Optimized Coordinates for the synthesis of 3-methyl-azetidine-2-one derivative by the reaction of a substituted aziridine with CO using the B3LYP DFT in conjunction with different basis sets.

Table S8: Optimized Coordinates of 3- methyl-azetidin-2-one (P*a*) and 4- methyl-azetidin-2-one (P*b*) products by the reaction of a methyl aziridine with CO at the B3LYP / 6-31+G (d) level in different solvents (CPCM).

Table S9: Optimized Coordinates of 3- methyl-azetidin-2-one (P*a*) and 4- methyl-azetidin-2-one (P*b*) derivatives by the reaction of a substituted aziridine with CO calculated by the reaction of a methyl aziridine with CO.

Table S10: Optimized Coordinates for cycloaddition reaction of CO with aziridine catalyzed by NaBr. (Page 12, Figure 3)

Table S11: Optimized Coordinates for cycloaddition reaction of CO with aziridine catalyzed by bilayer $Mg_{16}O_{16}$ cluster surface on Mg in the middle of the surface. (Page 16, Figure 5)

Table S12: Optimized Coordinates for cycloaddition reaction of CO with aziridine catalyzed bilayer $Mg_{16}O_{16}$ cluster surface on Mg at the surface end. (Page 16, Figure 6)

Figures

Figure S1: Comparison of the optimized structures of CO, 3-methyl- aziridine, 4-methyl- aziridine, 3-methyl-azetidin-2-one and 4-methyl-azetidin-2-one as computed at the B3LYP/6-31+G(d) (bold atoms) and CCSD(T)-F12/aug-cc-pVDZ (shadow atoms) levels.

Figure S2: Optimized structures of the Mg_8O_8 cluster (monolayer) at different level of theory. We give also the numbering of the atoms used in the present work.

Figure S3: Optimized structures of the $Mg_{16}O_{16}$ bilayer surface at the PBE/6-31+G(d) level. We give also the numbering of the atoms used in the present work.

Figure S4: Adsorption of CO on Mg_8O_8 monolayer crystal as optimized at the PBE / 6-31+G level.

Figure S5: Optimized structures of the adsorption of CO on $Mg_{16}O_{16}$ bilayer cluster for both (*a*) CO and (*b*) OC configurations as calculated at the PBE / 6-31+G level. We give also the numbering of the atoms used in the present work.

Figure S6: Optimized structures of the $Mg_{16}O_{16}$ bilayer surface and the transition state in azetidin-2-one synthesis (*i*) on Mg at mid-surface (*ii*) on Mg at cluster edge. We give also the numbering of the atoms used in the present work.

Benchmarks on the CO adsorption on $Mg_{16}O_{16}$: Carbon monoxide adsorbs on MgO through two possible interactions (O-Mg or C-Mg). The strength of these interactions can be evaluated *via* the determination of the adsorption energy, $E_{adsorption}$, expressed as follows:

$$E_{adsorption} = E_{CO@MgO\ cluster} - (E_{MgO\ cluster} + E_{CO}) \quad (1)$$

where $E_{CO@MgO\ cluster}$, $E_{MgO\ cluster}$ and E_{CO} correspond to the total energies of CO@MgO cluster, MgO cluster and CO, respectively as evaluated in the basis set of CO@MgO cluster. Alternatively, we applied a basis set superposition error (BSSE) correction factor (using the keyword Counter = n, where in this case n=2) to account for structural distortions occurring during intermolecular interactions between the MgO cluster and carbon monoxide.

Table: Adsorption energies ($E_{adsorption}$, in kcal.mol^{-1}) of CO on $Mg_{16}O_{16}$ bilayer cluster calculated at the PBE/6-31+G(d) level.

$E_{adsorption}$ / **Bond**	**Without dispersion correction**		**With dispersion correction**	
	**Without BSSE**	**With BSSE**	**Without BSSE**	**With BSSE**
**C=O**	-5.61	-3.97	-8.11	-6.45
**O=C**	-1.52	-0.46	-3.71	-2.29

The table above reveals that adsorption occurs through the interaction of the carbon atom of CO on a preferential site (Mg of the MgO surface) with an adsorption energy of -8.11 kcal.mol^{-1}, compared to -3.71 kcal.mol^{-1} for the interaction between the oxygen of CO and Mg. Despite these relatively low adsorption energies (in absolute values), significant deformation of the magnesium oxide monolayer was observed. In the case of the bilayer MgO surface, we analyzed both adsorption modes and found once again that carbon adsorption on Mg has the lowest adsorption energy with a nearly undistorted structure. Geometric parameters and RMSD values are summarized in Table S3. With the inclusion of dispersion correction, the adsorption energy of CO on the $Mg_{16}O_{16}$ bilayer enhances remarkably. Besides there is a notable reduction in the adsorption energy values for both CO and OC configurations, highlighting the significant impact of dispersion forces on the adsorption process. Therefore, the $Mg_{16}O_{16}$ bilayer is better suited to mimic the MgO (001). It will be investigated further to check its potentialities as a catalyst of the carbonylation reaction of aziridines.

Table S1:

Distance	B3LYP/6-31G(d)	B3LYP/6-31+G(d)	PBE/6-31+G(d)
Mg_1-O_4	1.883	1.892	1.912
Mg_1-O_3	1.975	1.98	1.992
Mg_1-O_{12}	1.981	1.986	1.997
Mg_2-O_4	1.883	1.892	1.912
Mg_2-O_3	1.975	1.98	1.992
Mg_2-O_8	1.981	1.986	1.997
Mg_5-O_3	1.924	1.93	1.946
Mg_5-O_8	2.064	2.068	2.082
Mg_5-O_7	2.064	2.068	2.082
Mg_5-O_{16}	1.924	1.93	1.946
Mg_6-O_8	1.848	1.855	1.873
Mg_6-O_7	1.848	1.855	1.873
Mg_9-O_{12}	1.848	1.855	1.873
Mg_9-O_{11}	1.848	1.855	1.873
Mg_{10}-O_{12}	2.064	2.068	2.082
Mg_{10}-O_3	1.924	1.93	1.946
Mg_{10}-O_{16}	1.924	1.93	1.946
Mg_{10}-O_{11}	2.064	2.068	2.082
Mg_{13}-O_{11}	1.981	1.986	1.997
Mg_{13}-O_{16}	1.975	1.98	1.992
Mg_{13}-O_{15}	1.883	1.892	1.912
Mg_{14}-O_{16}	1.975	1.98	1.992
Mg_{14}-O_7	1.981	1.986	1.992
Mg_{14}-O_{15}	1.883	1.892	1.912
**E_{Gap}**	1.745	1.808	0.465
**E_{Total}**	2546.952	2569.544	1465.027
**RMSD**	0.794451	0.765057	0.691776

Table S2:

Mg_1-O_7	1.968	Mg_{18}-O_{32}	2.017
Mg_1-O_6	1.968	Mg_{18}-O_{24}	2.096
Mg_1-O_8	1.941	Mg_{18}-O_5	2.017
Mg_2-O7	2.137	Mg_{18}-O_{21}	2.096
Mg_2-O_5	2.036	Mg_{19}-O_{22}	1.941
Mg_2-O_8	2.005	Mg_{19}-O_{24}	1.968
Mg_2-O_{16}	2.006	Mg_{19}-O_{21}	1.968
Mg_3-O_6	2.137	Mg_{20}-O_{22}	2.005
Mg_3-O_5	2.036	Mg^{20}-O_{30}	2.006
Mg_3-O_8	2.005	Mg_{20}-O_{23}	2.036
Mg_3-O_{24}	2.006	Mg_{20}-O_{21}	2.137
Mg_4-O_6	2.096	Mg_{25}-O_{14}	2.017
Mg_4-O_{14}	2.017	Mg_{25}-O_{30}	2.096
Mg_4-O_7	2.096	Mg_{25}-O_{31}	2.096
Mg_4-O_{23}	2.017	Mg_{25}-O_{23}	2.017
Mg_9-O_7	2.006	Mg_{26}-O_{13}	2.006
Mg_9-O_{15}	2.005	Mg_{26}-O_{29}	2.005
Mg_9-O_{16}	2.137	Mg_{26}-O_{32}	2.036
Mg_9-O_{14}	2.036	Mg_{26}-O_{31}	2.137
Mg_{10}-O_{16}	1.968	Mg_{27}-O_{29}	2.005
Mg_{10}-O_{15}	1.941	Mg_{27}-O_{21}	2.006
Mg_{10}-O_{13}	1.968	Mg_{27}-O_{32}	2.036
Mg_{11}-O_{16}	2.096	Mg_{27}-O_{30}	2.137
Mg_{11}-O_{32}	2.017	Mg_{28}-O_{29}	1.941
Mg_{11}-O_{13}	2.096	Mg_{28}-O_{31}	1.968
Mg_{11}-O_5	2.017	Mg_{28}-O_{30}	1.968
Mg_{12}-O_{13}	2.137	**RMSD**	0.593341
Mg_{12}-O_{31}	2.006		
Mg_{12}-O_{15}	2.005		
Mg_{12}-O_{14}	2.036		
Mg_{17}-O_{22}	2.005		
Mg_{17}-O_6	2.006		
Mg_{17}-O_{24}	2.137		
Mg_{17}-O_{23}	2.036		

Table S3:

Distance	CO configuration	OC configuration	Distance	CO configuration	OC configuration
Mg_1-O_7	1.971	1.968	Mg_{19}-O_{22}	1.942	1.941
Mg_1-O_6	1.972	1.968	Mg_{19}-O_{24}	1.969	1.968
Mg_1-O_8	1.934	1.939	Mg_{19}-O_{21}	1.967	1.968
Mg_2-O_7	2.144	2.137	Mg_{20}-O_{22}	2.006	2.006
Mg_2-O_5	2.028	2.034	Mg_{20}-O_{30}	2.007	2.006
Mg_2-O_8	2.006	2.006	Mg_{20}-O_{23}	2.033	2.035
Mg_2-O_{16}	2.004	2.006	Mg_{20}-O_{21}	2.136	2.136
Mg_3-O_6	2.147	2.136	Mg_{25}-O_{14}	2.018	2.017
Mg_3-O_5	2.027	2.034	Mg_{25}-O_{30}	2.098	2.096
Mg_3-O_8	2.005	2.006	Mg_{25}-O_{31}	2.097	2.096
Mg_3-O_{24}	2.004	2.006	Mg_{25}-O_{23}	2.018	2.017
Mg_4-O_6	2.114	2.099	Mg_{26}-O_{13}	2.006	2.006
Mg_4-O_{14}	2.022	2.019	Mg_{26}-O_{29}	2.006	2.006
Mg_4-O_7	2.114	2.099	Mg_{26}-O_{32}	2.036	2.037
Mg_4-O_{23}	2.023	2.019	Mg_{26}-O_{31}	2.138	2.138
Mg_9-O_7	2.013	2.006	Mg_{27}-O_{29}	2.006	2.006
Mg_9-O_{15}	2.006	2.006	Mg_{27}-O_{21}	2.006	2.006
Mg_9-O_{16}	2.128	2.135	Mg_{27}-O_{32}	2.036	2.037
Mg_9-O_{14}	2.037	2.037	Mg_{27}-O_{30}	2.138	2.138
Mg_{10}-O_{16}	1.969	1.968	Mg_{28}-O_{29}	1.941	1.941
Mg_{10}-O_{15}	1.941	1.941	Mg_{28}-O_{31}	1.968	1.968
Mg_{10}-O_{13}	1.967	1.968	Mg_{28}-O_{30}	1.968	1.968
Mg_{11}-O_{16}	2.099	2.097	**RMSD**	0.586369	0.591792
Mg_{11}-O_{32}	2.017	2.017			
Mg_{11}-O_{13}	2.101	2.098			
Mg_{11}-O_5	2.013	2.016			
Mg_{12}-O_{13}	2.136	2.136			
Mg_{12}-O_{31}	2.007	2.006			
Mg_{12}-O_{15}	2.007	2.006			
Mg_{12}-O_{14}	2.033	2.035			
Mg_{17}-O_{22}	2.005	2.006			
Mg_{17}-O_6	2.013	2.006			
Mg_{17}-O_{24}	2.126	2.135			
Mg_{17}-O_{23}	2.038	2.037			
Mg_{18}-O_{32}	2.016	2.017			
Mg_{18}-O_{24}	2.099	2.096			
Mg_{18}-O_5	2.012	2.016			
Mg_{18}-O_{21}	2.1	2.098			

Table S4:

Distance	Mg at mid-surface	Mg at cluster edge	Distance	Mg at mid-surface	Mg at cluster edge
Mg_1-O_7	1.975	1.967	Mg_{19}-O_{22}	1.948	1.946
Mg_1-O_6	1.963	1.963	Mg_{19}-O_{24}	1.969	1.967
Mg_1-O_8	1.924	1.942	Mg_{19}-O_{21}	1.96	1.96
Mg_2-O7	2.228	2.14	Mg_{20}-O_{22}	2.018	2.012
Mg_2-O_5	2.001	2.032	Mg_{20}-O_{30}	1.998	1.969
Mg_2-O_8	2.005	2.003	Mg_{20}-O_{23}	2.02	2.051
Mg_2-O_{16}	1.994	1.994	Mg_{20}-O_{21}	2.104	2.124
Mg_3-O_6	2.178	2.145	Mg_{25}-O_{14}	2.047	2.061
Mg_3-O_5	2.02	2.04	Mg_{25}-O_{30}	2.109	2.105
Mg_3-O_8	2.019	2.006	Mg_{25}-O_{31}	2.108	2.122
Mg_3-O_{24}	1.997	1.99	Mg_{25}-O_{23}	2.047	2.049
Mg_4-O_6	2.134	2.108	Mg_{26}-O_{13}	2.01	2.059
Mg_4-O_{14}	2.025	2.022	Mg_{26}-O_{29}	2.006	2.001
Mg_4-O_7	2.149	2.113	Mg_{26}-O_{32}	2.012	2.033
Mg_4-O_{23}	2.017	2.009	Mg_{26}-O_{31}	2.18	2.043
Mg_9-O_7	2.025	2.007	Mg_{27}-O_{29}	2.007	2.003
Mg_9-O_{15}	2	2.006	Mg_{27}-O_{21}	2.008	2.046
Mg_9-O_{16}	2.1	2.125	Mg_{27}-O_{32}	2.013	2.043
Mg_9-O_{14}	2.015	2.035	Mg_{27}-O_{30}	2.183	2.05
Mg_{10}-O_{16}	1.972	1.964	Mg_{28}-O_{29}	1.927	1.989
Mg_{10}-O_{15}	1.95	1.947	Mg_{28}-O_{31}	1.961	2.007
Mg_{10}-O_{13}	1.958	1.961	Mg_{28}-O_{30}	1.96	2.005
Mg_{11}-O_{16}	2.146	2.115	**RMSD**	0.562228	0.590888
Mg_{11}-O_{32}	2	1.992			
Mg_{11}-O_{13}	2.124	2.071			
Mg_{11}-O_5	2.019	2.016			
Mg_{12}-O_{13}	2.106	2.12			

Mg_{12}-O_{31}	1.995	1.958			
Mg_{12}-O_{15}	2.02	2.013			
Mg_{12}-O_{14}	2.023	2.058			
Mg_{17}-O_{22}	2.003	2.002			
Mg_{17}-O_{6}	2.02	2.01			
Mg_{17}-O_{24}	2.115	2.129			
Mg_{17}-O_{23}	2.023	2.032			
Mg_{18}-O_{32}	2.002	1.998			
Mg_{18}-O_{24}	2.136	2.119			
Mg_{18}-O_{5}	2.024	2.028			
Mg_{18}-O_{21}	2.125	2.078			

Table S5:
Optimized Coordinates at the PBE/6-31+G(d) level of theory

16 atoms, **CO@aziridine [CH_3, CH_3] (Table 1)**
PBE/6-31+G(d). E = -325.462523886 H

C	-0.9424807481	1.9665539955	1.2823439519
C	0.6320383697	-1.1723579313	0.9464322491
C	1.2427907877	-0.781636739	-0.3661465675
H	0.4482731663	-2.241420321	1.1297423656
H	0.8307164645	-0.5725965665	1.8438693629
H	1.4321207509	-1.6207157762	-1.056928693
O	-2.0214387439	2.1408406973	0.9213055883
C	2.1770882047	0.3984544281	-0.5143319877
H	3.2246016022	0.0897629193	-0.3453080985
H	2.1084702713	0.8336726195	-1.5278285473
H	1.9228831726	1.1862798701	0.2138861748
C	-1.1563854106	-1.3364772349	-0.7784909615
H	-2.0939862497	-1.3504263387	-0.1954288631
H	-1.3697350514	-0.8969969283	-1.7684163993
H	-0.8205662392	-2.3889260272	-0.9257781282
N	-0.173635347	-0.5056586667	-0.0868164465

16 atoms, **P [CH_3, CH_3]**
PBE/6-31+G(d). E = -325.527202776 H

C	-0.2294582282	0.5070290707	-0.5636480237
C	0.696513182	-1.317645479	-0.0478958348
C	1.2515014519	0.0395116562	-0.5905004948
H	0.8253688589	-2.1928633849	-0.7122108063
H	1.025883289	-1.5758188247	0.977045191
H	1.6281275273	-0.0428019113	-1.6258726221
O	-0.8379541948	1.5388730907	-0.8239234553
C	2.2243210924	0.8209631337	0.2870889284
H	3.2218632155	0.3473756924	0.3005197633
H	2.3391168798	1.850745836	-0.092309526
H	1.8590072402	0.8850837993	1.3277069788
C	-1.951819005	-1.2448094143	0.2369064223
H	-2.0038358474	-1.5413768423	1.3015623293
H	-2.6884121514	-0.4460691474	0.0530308694
H	-2.210082898	-2.120770538	-0.3876425451
N	-0.6479174121	-0.7252057371	-0.100761174

16 atoms, **TS [CH_3, CH_3]**
PBE/6-31+G(d). E = -325.397228357 H

C	-1.3387117183	0.8894940635	1.169940233
C	0.5459525588	-0.8011874561	0.9614745444
C	1.5020165147	-0.8122257243	-0.1533153451
H	0.231927425	-1.8025340083	1.3177761529
H	0.8095186149	-0.1578962166	1.829641454
H	1.577559544	-1.7160318168	-0.7726973081
O	-2.257680109	1.4706138598	0.6836935731
C	2.3379701693	0.3589873034	-0.5277495111
H	3.4145904375	0.0888140976	-0.4674922716
H	2.1706396991	0.6825766593	-1.573839741
H	2.1556418288	1.2223386879	0.1319958664
C	-1.307721895	-0.8854880714	-0.7358466995
H	-1.3676408035	-1.9479522677	-0.4303027187

H -2.3218938388 -0.4552506245 -0.7344714553
H -0.9209686016 -0.8262794896 -1.7647359675
N -0.475093826 -0.0877889962 0.2095071939

Optimized Coordinates at the B3LYP/6-31+G(d) level of theory
16 atoms, **CO@aziridine [CH_3, CH_3] (Table 1)**
B3LYP/6-31+G(d). E = -325.877695747 H
C -1.067217431 2.1681210316 1.3701203898
C 0.6402853471 -1.1806874279 0.9423988244
C 1.255967144 -0.8087258537 -0.3671569959
H 0.4577291638 -2.2388483509 1.1384342292
H 0.8319009422 -0.5769699216 1.8280800998
H 1.4523688289 -1.6486149747 -1.0403043202
O -2.1139698005 2.2989827005 0.9424539554
C 2.1902371401 0.3683455383 -0.5278099663
H 3.2304234851 0.0639604215 -0.3551215584
H 2.1225181283 0.787374829 -1.5398500431
H 1.9377238336 1.1602206933 0.1846791339
C -1.1339759636 -1.3707334791 -0.788524829
H -2.0673413866 -1.3794501638 -0.2142115685
H -1.3416597296 -0.9461808762 -1.7771941602
H -0.798005312 -2.4156091788 -0.9209833716
N -0.1562293897 -0.5328329877 -0.1029048193
16 atoms, **P [CH_3, CH_3]**
B3LYP/6-31+G(d). E = -325.931849972 H
C -0.227598975 0.4981811966 -0.5618552833
C 0.6972275527 -1.3189515314 -0.0456177732
C 1.2495985487 0.0378199441 -0.5887948336
H 0.8262682063 -2.1870474765 -0.7044881268
H 1.0219454027 -1.5755787423 0.9714895674
H 1.6244267025 -0.0426598342 -1.6158000619
O -0.8305195162 1.5223402697 -0.8220165641
C 2.220519525 0.8221816743 0.2867615364
H 3.209555565 0.3487141014 0.3026546291
H 2.337245024 1.8424343452 -0.0949635849
H 1.8572716276 0.890333741 1.319472494
C -1.9501033708 -1.2399509834 0.2349494339
H -2.0025425063 -1.5305143015 1.292612257
H -2.6812022272 -0.448216258 0.0489914778
H -2.2051102128 -2.1113678262 -0.3824946089
N -0.6447583462 -0.7254973186 -0.101804559
16 atoms, **TS [CH_3, CH_3]**
B3LYP/6-31+G(d). E = - 325.7852786 H
C -0.653320 1.080677 0.409732
C 0.631842 -1.047379 0.723788
C 1.489679 -0.527622 -0.349019
H 0.519548 -2.135062 0.744022
H 0.823994 -0.647901 1.720811
H 1.426571 -1.056369 -1.297520
O -1.621645 1.594842 -0.093453
C 2.489085 0.573551 -0.240757
H 3.487293 0.146340 -0.451123
H 2.331316 1.360158 -0.987170
H 2.489600 1.032672 0.749223
C -1.635632 -1.247219 -0.414095

H	-2.156121	-1.745137	0.416104
H	-2.329233	-0.588104	-0.940382
H	-1.259635	-2.012345	-1.101889
N	-0.521090	-0.414452	0.078265

Optimized Coordinates at the M06-2X/6-31+G(d) level of theory

16 atoms, **CO@aziridine [CH_3, CH_3] (Table 1)**

M06-2X /6-31+G(d). E = -325.7299385 H

C	-0.57310000	2.13381600	0.87280700
C	0.61009500	-1.80482400	0.58738900
C	1.00423500	-0.88232300	-0.51382700
H	0.30986200	-2.81741000	0.31800100
H	1.06596600	-1.70047700	1.56904700
H	0.93773500	-1.30433600	-1.51940400
O	-1.24380900	2.22217400	-0.03634900
C	2.04627500	0.18987400	-0.32449200
H	3.04597700	-0.19480000	-0.55167700
H	1.85098100	1.04563300	-0.98021400
H	2.03476800	0.54414900	0.71063000
C	-1.45495900	-1.09467500	-0.55684400
H	-2.24443400	-1.36084500	0.15225900
H	-1.79417800	-0.24206200	-1.15316200
H	-1.28568000	-1.95127200	-1.23133300
N	-0.26029900	-0.71522900	0.17899000

16 atoms, **P [CH_3, CH_3]**

M06-2X /6-31+G(d). E = -325.7753697 H

C	-0.22048600	0.62596100	-0.23008900
C	0.46619400	-1.34575300	-0.09250300
C	1.16369000	-0.00539800	-0.46266500
H	0.41948700	-2.09569300	-0.88994900
H	0.82711500	-1.80675500	0.83512800
H	1.46071600	0.04433800	-1.51476400
O	-0.68532000	1.74223400	-0.26835300
C	2.26628100	0.49176900	0.45698800
H	3.18183900	-0.09455100	0.33065900
H	2.49443600	1.53950300	0.24141400
H	1.95455500	0.42352000	1.50521400
C	-2.11135200	-0.97102000	0.42938600
H	-2.14111800	-1.43206600	1.42347800
H	-2.72180600	-0.06484500	0.43886300
H	-2.52616000	-1.67372400	-0.30285700
N	-0.77052100	-0.58607400	0.07527500

16 atoms, **TS [CH_3, CH_3]**

M06-2X /6-31+G(d). E = -325.6210626 H

C	-0.32079500	1.06866200	0.45935400
C	0.44148500	-1.25165800	0.62659200
C	1.10292200	-0.43400500	-0.39493000
H	0.22991300	-2.28953000	0.37257500
H	0.77063200	-1.12397300	1.65605700
H	0.86176300	-0.73440000	-1.41433500
O	-1.14073300	1.83582300	0.00848000
C	2.34379500	0.39076300	-0.24000500
H	3.18389000	-0.20131200	-0.64077500
H	2.28052600	1.31767500	-0.81272000
H	2.52938700	0.63615200	0.80454900

C	-1.84590200	-0.88469300	-0.43815700
H	-2.47206500	-1.46795600	0.24528200
H	-2.39347900	-0.01190700	-0.80239900
H	-1.55011800	-1.52149900	-1.28034700
N	-0.66337400	-0.37489300	0.24673500

Optimized Coordinates at the ωB97XD/6-31+G(d) level of theory

16 atoms, **CO@aziridine [CH_3, CH_3] (Table 1)**

ωB97XD/6-31+G(d). E = -325.7710514 H

C	-0.72997500	2.13934100	0.85019500
C	0.68495600	-1.71880800	0.70031300
C	1.10004100	-0.90521400	-0.47728800
H	0.44376800	-2.76943300	0.53319200
H	1.07887400	-1.48820400	1.68778500
H	1.10609300	-1.43912400	-1.43108600
O	-1.45420400	2.25100700	-0.01878100
C	2.08992200	0.22671300	-0.37135300
H	3.11254400	-0.13945800	-0.51684900
H	1.88937300	0.99214600	-1.12969200
H	2.02600600	0.69892500	0.61377000
C	-1.35482900	-1.21760100	-0.60783600
H	-2.16704300	-1.43358900	0.09354000
H	-1.69502400	-0.44433800	-1.30402400
H	-1.13770200	-2.13537700	-1.18287800
N	-0.20800700	-0.72020900	0.13047500

16 atoms, **P [CH_3, CH_3]**

ωB97XD/6-31+G(d). E = -325.8289084 H

C	-0.24632600	0.61572800	-0.23578600
C	0.51673400	-1.32486000	-0.05994900
C	1.16434000	0.03566600	-0.44702700
H	0.50840300	-2.08984300	-0.84525700
H	0.88473700	-1.75869200	0.87830300
H	1.46126000	0.07788000	-1.49991300
O	-0.75729400	1.71277800	-0.30125900
C	2.25575200	0.58751100	0.45538200
H	3.18888600	0.02759100	0.33140000
H	2.44974900	1.63742400	0.21554200
H	1.95855800	0.53215000	1.50885700
C	-2.07399900	-1.05656900	0.42159500
H	-2.09474300	-1.51157600	1.41892400
H	-2.73135100	-0.18348800	0.41640700
H	-2.44437400	-1.78531600	-0.30943600
N	-0.74981900	-0.61296800	0.08289300

16 atoms, **TS [CH_3, CH_3]**

ωB97XD/6-31+G(d). E = -325.6703343 H

C	-0.41862600	1.08464900	0.43636300
C	0.48378100	-1.18103800	0.65632200
C	1.20215400	-0.44922900	-0.38793300
H	0.30692600	-2.24119100	0.47012300
H	0.77453500	-0.98552400	1.68729700
H	1.00522000	-0.80802500	-1.39640900
O	-1.28964300	1.79678200	-0.01405600
C	2.37449800	0.45986400	-0.22776800
H	3.26736800	-0.09382500	-0.56737800
H	2.28315900	1.35070400	-0.85307600

H	2.50539100	0.77226800	0.80765600
C	-1.81310000	-0.98035000	-0.42254800
H	-2.39562600	-1.54889300	0.31148300
H	-2.41596800	-0.16309900	-0.82600500
H	-1.50828300	-1.65272400	-1.23282000
N	-0.63968800	-0.37247400	0.19785200

Table S6:
Optimized Coordinates at the B3LYP/6-31G(d) level of theory
16 atoms, **CO@aziridine [CH_3, CH_3] (Table 2)**
B3LYP/6-31G(d). E = -325.862025 H

C	-0.82784600	1.97100400	0.67284700
C	0.75255400	-1.50025100	0.86520000
C	1.20000400	-0.82421800	-0.38971100
H	0.59192200	-2.57890500	0.82949800
H	1.06246900	-1.11781000	1.83642000
H	1.30014200	-1.48499900	-1.25524300
O	-1.69287800	1.97706500	-0.07182900
C	2.11672100	0.37766500	-0.39484700
H	3.16707700	0.06342100	-0.41679900
H	1.93477500	1.00665000	-1.27473000
H	1.95371100	0.98996000	0.49665100
C	-1.24602500	-1.28773000	-0.61059400
H	-2.07582800	-1.48056200	0.07859200
H	-1.60400600	-0.61184300	-1.39472100
H	-0.95558500	-2.24432800	-1.07954600
N	-0.16058300	-0.64404700	0.11451600

21 atoms, **P [CH_3, CH_3]**
B3LYP/6-31G(d). E = -325.9135914 H

C	-0.30634400	0.60669400	-0.13168400
C	0.59460000	-1.25956500	0.22375000
C	1.13174000	0.07885100	-0.37032200
H	0.65163000	-2.13001300	-0.44231500
H	0.99393900	-1.52849500	1.20929600
H	1.23966700	-0.03708300	-1.45811800
O	-0.89929800	1.65047600	-0.28977700
C	2.35675700	0.78517300	0.18466600
H	3.26889200	0.22175300	-0.04459800
H	2.45290400	1.77784800	-0.26672900
H	2.29884600	0.91382000	1.27114300
C	-2.03648800	-1.24578600	0.10936300
H	-2.35459700	-1.81126100	0.99294800
H	-2.75537500	-0.44236900	-0.07360200
H	-2.03410000	-1.92467600	-0.75692100
N	-0.73677800	-0.63989800	0.29015300

21 atoms, **TS [CH_3, CH_3]**
B3LYP/6-31G(d). E = -325.7663503H

C	-0.59742500	1.13417800	0.39187200
C	0.55017200	-1.04396000	0.71409400
C	1.40151600	-0.49542900	-0.34643700
H	0.42391300	-2.12995100	0.69156700
H	0.75082300	-0.68826600	1.72512600
H	1.32298200	-1.00148500	-1.30561700
O	-1.55801200	1.70911600	-0.05396500
C	2.42103100	0.58237500	-0.22296800
H	3.41797400	0.14663700	-0.42489900
H	2.28033100	1.37518400	-0.96533300
H	2.41591600	1.03692800	0.76809200
C	-1.75105100	-1.14567600	-0.39024000
H	-2.26312800	-1.67318500	0.42635300
H	-2.43361200	-0.42714100	-0.84930000
H	-1.43621100	-1.88301800	-1.13714800

N	-0.59433600	-0.37393700	0.08928000

Optimized Coordinates at the B3LYP/6-311++G (d,p) level of theory

16 atoms, **CO@aziridine [CH_3, CH_3] (Table 2)**

B3LYP/6-311++G (d,p). E = -325.9606004 H

C	-0.99893200	1.93518900	0.65718000
C	0.80846600	-1.39802800	0.92227300
C	1.27378600	-0.78546800	-0.35845400
H	0.68412000	-2.47881300	0.95128700
H	1.07225400	-0.94061500	1.87129600
H	1.42583000	-1.49284200	-1.17502700
O	-1.85002800	1.87971700	-0.08595500
C	2.14823900	0.44432100	-0.40658400
H	3.20636300	0.16531500	-0.38309500
H	1.97054900	1.01581900	-1.32295100
H	1.94277500	1.09685800	0.44429900
C	-1.14333100	-1.36176400	-0.62881600
H	-1.99432300	-1.52640300	0.03754900
H	-1.48655600	-0.76467500	-1.47851800
H	-0.80191100	-2.34024800	-1.00444200
N	-0.10778000	-0.62933800	0.08562000

21 atoms, **P [CH_3, CH_3]**

B3LYP/6-311++G(d,p). E = -326.0110238 H

C	-0.32541200	0.58433600	-0.17162700
C	0.65394600	-1.24025500	0.19101500
C	1.13655000	0.12890700	-0.38235900
H	0.74678300	-2.09371400	-0.48775100
H	1.05966100	-1.50362700	1.17238200
H	1.29226100	0.03544500	-1.46295400
O	-0.95650400	1.59976100	-0.34298400
C	2.29134200	0.89310000	0.24140800
H	3.24287700	0.38854500	0.05041900
H	2.35216700	1.89643300	-0.18764700
H	2.17123400	0.99749100	1.32309600
C	-1.99210500	-1.31828600	0.20557700
H	-2.20531100	-1.86707700	1.12779300
H	-2.75198700	-0.54676100	0.07120500
H	-2.04279200	-2.01606500	-0.63869200
N	-0.70012500	-0.66968200	0.25584400

21 atoms, **TS [CH_3, CH_3]**

B3LYP/6-311++G(d,p). E = -325.8679318 H

C	-0.74391200	1.11385100	0.40640700
C	0.59307200	-0.96274200	0.73208100
C	1.47820000	-0.46897400	-0.32740000
H	0.48978600	-2.04899400	0.76874800
H	0.77546300	-0.55248000	1.72373500
H	1.42038000	-1.00610700	-1.26896700
O	-1.70549500	1.62577400	-0.09188700
C	2.45686300	0.64150800	-0.22906800
H	3.46332200	0.23307500	-0.42431100
H	2.29451600	1.40841600	-0.99165500
H	2.44006100	1.11983300	0.74870600
C	-1.67480700	-1.21728800	-0.39442400
H	-2.18900500	-1.72249500	0.43166700
H	-2.38002700	-0.58204600	-0.92878300

H	-1.27962900	-1.97560000	-1.07473700
N	-0.57819900	-0.35970400	0.08930300

Optimized Coordinates at the B3LYP/6-311++G (2d,2p) level of theory

16 atoms, **CO@aziridine [CH_3, CH_3] (Table 2)**

B3LYP/6-311++G (2d,2p). E = -325.9726919 H

C	-1.04170800	2.03565900	0.67360600
C	0.82889200	-1.44481800	0.90474200
C	1.28116500	-0.81491000	-0.36834700
H	0.69977700	-2.52195400	0.91795900
H	1.10545600	-1.00409600	1.85432500
H	1.42243900	-1.50690900	-1.19537800
O	-1.87522800	1.95393000	-0.08229800
C	2.15849900	0.40995300	-0.40389200
H	3.21332300	0.12908800	-0.39289600
H	1.97644400	0.99543400	-1.30700500
H	1.96250000	1.04767400	0.45685900
C	-1.12676700	-1.39309300	-0.62738700
H	-1.97987300	-1.55314600	0.03228300
H	-1.46241000	-0.80220500	-1.48014200
H	-0.78560600	-2.37127500	-0.99444400
N	-0.09500500	-0.65933800	0.08932300

21 atoms, **P [CH_3, CH_3]**

B3LYP/6-311++G(2d,2p). E = -326.0191611 H

C	-0.33028900	0.59239200	-0.13118200
C	0.63946800	-1.22609400	0.25755300
C	1.12156600	0.11787300	-0.36354800
H	0.72352900	-2.09935400	-0.39236500
H	1.05024800	-1.45862000	1.24125900
H	1.21692600	-0.01653800	-1.44499900
O	-0.96004900	1.60437800	-0.31406300
C	2.32625900	0.87299200	0.15875800
H	3.24660000	0.32843500	-0.05846200
H	2.39512500	1.84708400	-0.32285600
H	2.27171000	1.03347000	1.23530900
C	-1.97876300	-1.32381800	0.09777400
H	-2.28648000	-1.91083100	0.96311200
H	-2.73391300	-0.56555700	-0.09711700
H	-1.91358500	-1.98806500	-0.76988700
N	-0.71579300	-0.65495200	0.32015900

21 atoms, **TS [CH_3, CH_3]**

B3LYP/6-311++G(2d,2p). E = -325.8783189 H

C	-0.72699600	1.11504600	0.40354500
C	0.58875700	-0.96998100	0.72806500
C	1.46465300	-0.46739800	-0.33065900
H	0.48562900	-2.05337500	0.75577100
H	0.77633900	-0.56962300	1.71933600
H	1.39995000	-0.99550200	-1.27334100
O	-1.68790200	1.63244400	-0.08743100
C	2.45085100	0.63294100	-0.22749000
H	3.45062700	0.21678800	-0.42560900
H	2.29544700	1.40340500	-0.98358300
H	2.43967900	1.10367100	0.75071000
C	-1.67994900	-1.20896800	-0.39334100
H	-2.18923200	-1.71979200	0.42824200

H	-2.38410900	-0.56737000	-0.91570600
H	-1.29273200	-1.96024500	-1.08149500
N	-0.58032600	-0.36104900	0.09205800

Table S7: Optimized Coordinates for noncatalyzed cycloaddition reaction of CO with aziridine calculated at the B3LYP/ 6-31+G(d) level in the gas phase.

__Path *b*__

16 atoms, **CO@aziridine [CH_3, CH_3] (Figure2)**

B3LYP/6-31+G(d). E = -325.8775542 H

C	-1.636576	-2.282302	-0.522854
C	1.355630	0.552927	0.019558
C	0.654099	0.136621	1.271311
H	1.772871	1.564197	0.038484
H	0.629855	0.843937	2.102319
O	-2.628989	-1.894019	-0.922970
C	-0.834183	1.738317	0.088110
H	-0.986120	2.085604	-0.940070
H	-1.816675	1.578769	0.546734
H	-0.312923	2.533803	0.651723
N	-0.104597	0.475241	0.064415
H	0.629801	-0.912529	1.561682
C	2.090360	-0.422780	-0.870566
H	2.078149	-0.086336	-1.915102
H	3.137666	-0.520264	-0.557340
H	1.621940	-1.411304	-0.827239

16 atoms, **P*b* [CH_3, CH_3]**

B3LYP/6-31+G(d). E = -325.9339296 H

C	-0.805774	-0.545946	0.510991
C	1.122610	0.285154	0.289131
C	0.584860	-0.994339	1.002223
H	1.478510	1.049190	0.995713
H	0.691525	-1.014537	2.090394
O	-1.949119	-0.944149	0.625449
C	-0.888745	1.653661	-0.814474
H	-0.496733	1.764325	-1.833095
H	-1.962967	1.456526	-0.870103
H	-0.729187	2.594937	-0.270431
N	-0.270450	0.543054	-0.130405
H	0.929326	-1.942509	0.578361
C	2.125275	0.097625	-0.841934
H	2.270924	1.027593	-1.405224
H	3.100276	-0.202381	-0.439019
H	1.786515	-0.676791	-1.540592

16 atoms, **TS*b* [CH_3, CH_3]**

B3LYP/6-31+G(d). E = -325.7832734 H

C	-0.823005	-0.757727	0.087646
C	1.167925	0.369729	0.158665
C	0.987867	-0.379155	1.438072
H	1.531284	1.402490	0.280923
H	0.483943	0.163073	2.237114
O	-1.965785	-1.075891	-0.146904
C	-1.013384	1.665511	-0.334468
H	-0.905362	2.146461	-1.313401
H	-2.068275	1.436118	-0.169169
H	-0.673631	2.365555	0.440744
N	-0.248969	0.433884	-0.212252
H	1.673542	-1.161437	1.757527
C	2.024735	-0.365130	-0.888493
H	1.878797	0.065315	-1.885177

H	3.089653	-0.298993	-0.635133
H	1.737734	-1.421954	-0.925320

Table S8:

Path *a*

Optimized Coordinates at the B3LYP/6-31+G(d)/CPCM (methanol) level

16 atoms, **CO@aziridine [CH_3, CH_3] (Table 3)**

B3LYP/6-31+G(d). E = -325.8805391 H

C	-1.34827500	2.40620200	0.87334800
C	0.95811700	-1.74675300	0.74126600
C	1.32773100	-0.94206700	-0.46076100
H	0.78206300	-2.81500600	0.60891500
H	1.33206200	-1.46286700	1.72372400
H	1.36826800	-1.50161100	-1.39878700
O	-1.97802000	2.36964100	-0.07334900
C	2.25128600	0.25243500	-0.39376900
H	3.29418000	-0.05832400	-0.53477200
H	2.00739600	0.98171700	-1.17680600
H	2.17080100	0.75289200	0.57745000
C	-1.10913800	-1.43193600	-0.60363400
H	-1.91923100	-1.67629200	0.09287000
H	-1.49026000	-0.71743100	-1.34186100
H	-0.81633300	-2.35543300	-1.13250900
N	-0.00630200	-0.81819600	0.13530200

16 atoms, **P*a* [CH_3, CH_3]**

B3LYP/6-31+G(d). E = -325.9420277 H

C	-0.29771800	0.58116500	-0.24549800
C	0.63372500	-1.28006500	-0.01333100
C	1.16380700	0.12709100	-0.43524400
H	0.69544200	-2.06292400	-0.77709400
H	1.01717100	-1.65398900	0.94299200
H	1.45373200	0.15986600	-1.49188000
O	-0.90931400	1.64373600	-0.34773000
C	2.21886500	0.79573000	0.43952400
H	3.18934800	0.30009100	0.32180100
H	2.34162500	1.84795300	0.15815700
H	1.94112900	0.75609900	1.49985500
C	-1.99342700	-1.23271700	0.42340700
H	-1.98387900	-1.67013000	1.42901400
H	-2.73825400	-0.43335800	0.39022100
H	-2.27173600	-2.01026500	-0.29855900
N	-0.70213400	-0.66993700	0.09621200

16 atoms, **TS*a* [CH_3, CH_3]**

B3LYP/6-31+G(d). E = -325.8043707 H

C	-0.63064100	1.08509800	0.41521200
C	0.57824900	-1.04492700	0.69454900
C	1.36366700	-0.41074900	-0.36980000
H	0.46401000	-2.12677800	0.60418800
H	0.83225600	-0.75747400	1.71435500
H	1.19181900	-0.81036300	-1.36663400
O	-1.60982500	1.66604800	-0.06023700
C	2.44885900	0.58604200	-0.22044700
H	3.38449200	0.05701000	-0.48449300
H	2.35984100	1.41195700	-0.93324500
H	2.53308500	0.97600600	0.79402100
C	-1.70375800	-1.14277000	-0.41999400
H	-2.24640700	-1.70435100	0.35074100
H	-2.38149500	-0.45103500	-0.92234100

H	-1.29451600	-1.85164000	-1.14658700
N	-0.61467700	-0.35826800	0.18211000

Optimized Coordinates at the B3LYP/6-31+G(d)/CPCM (dichloro-methane) level

16 atoms, **CO@aziridine [CH_3, CH_3] (Table 3)**

B3LYP/6-31+G (d). E = -325.8799915 H

C	-1.23447200	2.28795300	0.85191100
C	0.90428800	-1.67361300	0.77838900
C	1.29973400	-0.91518800	-0.44543800
H	0.73114300	-2.74637000	0.68296400
H	1.25737900	-1.35239000	1.75708900
H	1.36030100	-1.51049100	-1.36025000
O	-1.91003900	2.28500900	-0.06406800
C	2.22317600	0.28068000	-0.40423000
H	3.26811700	-0.03556900	-0.51470600
H	1.99461200	0.98170200	-1.21709500
H	2.12528100	0.81576600	0.54654300
C	-1.13397300	-1.41059900	-0.61936200
H	-1.95665600	-1.63101500	0.07036100
H	-1.50228500	-0.72361200	-1.38955100
H	-0.83073500	-2.35222100	-1.10938900
N	-0.04610500	-0.76930800	0.11724100

21 atoms, **P*a* [CH_3, CH_3]**

B3LYP/6-31+G(d). E = -325.9409227 H

C	-0.29660100	0.58232600	-0.24464000
C	0.63031800	-1.28120300	-0.00428500
C	1.16396700	0.12256500	-0.43329500
H	0.69145900	-2.06858500	-0.76376300
H	1.01365400	-1.65080000	0.95396400
H	1.45096200	0.14894400	-1.49097700
O	-0.90493600	1.64457300	-0.35068800
C	2.22360100	0.79288500	0.43435000
H	3.19254700	0.29390600	0.31724500
H	2.34815600	1.84302900	0.14630200
H	1.94856300	0.76108500	1.49565600
C	-1.99895300	-1.22791800	0.41817700
H	-1.99958300	-1.66710500	1.42320100
H	-2.74061400	-0.42580200	0.38024100
H	-2.27463900	-2.00343700	-0.30733100
N	-0.70392900	-0.66814300	0.10174200

21 atoms, **TS*a* [CH_3, CH_3]**

B3LYP/6-31+G(d). E = -325.8020503 H

C	-0.62572400	1.08807000	0.41641600
C	0.57493300	-1.04837200	0.69571800
C	1.36253000	-0.41732600	-0.36901200
H	0.45765000	-2.13024600	0.60785300
H	0.82619500	-0.75840600	1.71557400
H	1.19629900	-0.82369500	-1.36409100
O	-1.59945900	1.67160400	-0.05966000
C	2.44545600	0.58395600	-0.22123400
H	3.38505600	0.06182200	-0.48471400
H	2.35071400	1.41066300	-0.93245900
H	2.52593600	0.97607800	0.79276000
C	-1.70897400	-1.13975400	-0.41896800
H	-2.25158700	-1.69795900	0.35441500

H	-2.38507400	-0.44397500	-0.91816600
H	-1.30680600	-1.85111800	-1.14726500
N	-0.61314700	-0.35934800	0.17655100

Optimized Coordinates at the B3LYP/6-31+G(d)/CPCM (diethyl ether) level

16 atoms, **CO@aziridine [CH_3, CH_3] (Table 3)**

B3LYP/6-31+G (d). E = -325.8795135 H

C	-1.18266700	2.27260600	0.83340800
C	0.88261500	-1.65092300	0.79656300
C	1.29008200	-0.91339000	-0.43637000
H	0.71349500	-2.72581200	0.71781900
H	1.22367400	-1.31181100	1.77347400
H	1.36249200	-1.52544600	-1.33939500
O	-1.88980700	2.26800600	-0.05863300
C	2.21015400	0.28542800	-0.40664500
H	3.25728400	-0.03003500	-0.49774400
H	1.99003000	0.97016000	-1.23560200
H	2.09820700	0.83860400	0.53204900
C	-1.14151000	-1.41485300	-0.62645600
H	-1.97048500	-1.62404800	0.05921300
H	-1.50311700	-0.74084100	-1.41115200
H	-0.83355800	-2.36460000	-1.09810300
N	-0.06136000	-0.76096300	0.10921000

21 atoms, **P*a* [CH_3, CH_3]**

B3LYP/6-31+G(d). E = -325.9393904 H

C	-0.29479400	0.58393600	-0.24978500
C	0.62690200	-1.28382000	-0.01021200
C	1.16549200	0.11939900	-0.43510200
H	0.68702900	-2.06987100	-0.77160000
H	1.01000900	-1.65746800	0.94692300
H	1.45955400	0.14899600	-1.49071400
O	-0.89912600	1.64598000	-0.35531300
C	2.21946700	0.78535400	0.44299100
H	3.18978400	0.28763000	0.33107100
H	2.34474400	1.83705300	0.16135800
H	1.93630700	0.74873900	1.50205800
C	-1.99936900	-1.21873300	0.42949100
H	-1.99376400	-1.64967800	1.43840000
H	-2.73639000	-0.41224000	0.39192100
H	-2.28742000	-1.99854900	-0.28680800
N	-0.70514600	-0.66776100	0.09666000

21 atoms, **TS*a* [CH_3, CH_3]**

B3LYP/6-31+G(d). E = -325.7988549 H

C	-0.62078200	1.09244400	0.41711500
C	0.57093000	-1.05111400	0.69825700
C	1.36356200	-0.42739300	-0.36738200
H	0.45025800	-2.13334800	0.61651000
H	0.81763400	-0.75488100	1.71755900
H	1.20621800	-0.84503200	-1.35929000
O	-1.58807700	1.67817500	-0.05921900
C	2.44121000	0.58228400	-0.22245100
H	3.38740500	0.07214800	-0.48554900
H	2.33630300	1.40968400	-0.93157600
H	2.51558400	0.97762900	0.79088500
C	-1.71537700	-1.13716000	-0.41728300

H	-2.26105600	-1.68576800	0.36109000
H	-2.38703700	-0.43787900	-0.91803800
H	-1.32203300	-1.85641100	-1.14294300
N	-0.61084600	-0.36084300	0.16793800

Optimized Coordinates at the B3LYP/6-31+G(d)/CPCM (toluene) level

16 atoms, **CO@aziridine [CH_3, CH_3] (Table 3)**

B3LYP/6-31+G (d). E = -325.8788297 H

C	-1.10662700	2.21349200	0.80445000
C	0.84921900	-1.60293300	0.82483800
C	1.27696500	-0.90114700	-0.42247200
H	0.68453400	-2.68039500	0.77438100
H	1.17167500	-1.23449000	1.79742500
H	1.36729200	-1.54035500	-1.30510200
O	-1.86176800	2.21941100	-0.04774900
C	2.19241500	0.30149800	-0.41202000
H	3.24262200	-0.01206500	-0.46706200
H	1.98978300	0.95687800	-1.26865600
H	2.05423200	0.88575300	0.50383700
C	-1.15229800	-1.40909800	-0.63720300
H	-1.99113800	-1.59905900	0.04204300
H	-1.50241300	-0.75599200	-1.44447800
H	-0.83876800	-2.37166600	-1.07899300
N	-0.08296900	-0.73646900	0.09548800

21 atoms, **P*a* [CH_3, CH_3]**

B3LYP/6-31+G(d). E = -325.93726 H

C	-0.29470200	0.58525900	-0.24899900
C	0.62539400	-1.28423800	-0.00016800
C	1.16553600	0.11622600	-0.43288000
H	0.68701000	-2.07515400	-0.75703500
H	1.00943700	-1.65334100	0.95880700
H	1.45579500	0.13917300	-1.48983700
O	-0.89697500	1.64465400	-0.35906500
C	2.22370700	0.78675700	0.43618300
H	3.19464300	0.29040900	0.32205700
H	2.34487300	1.83730600	0.14888800
H	1.94605600	0.75581200	1.49690300
C	-2.00362200	-1.21674000	0.42276700
H	-2.01056700	-1.64935500	1.43131300
H	-2.73704200	-0.40715000	0.37908400
H	-2.28859200	-1.99462700	-0.29757400
N	-0.70579700	-0.66783300	0.10288400

21 atoms, **TS*a* [CH_3, CH_3]**

B3LYP/6-31+G(d). E = -325.7947085 H

C	-0.62282100	1.09883300	0.41615100
C	0.56816900	-1.04814800	0.70318000
C	1.37196400	-0.44015200	-0.36361300
H	0.44518100	-2.13148500	0.63486100
H	0.80615300	-0.73848300	1.72079200
H	1.22931200	-0.87635800	-1.34984000
O	-1.58499500	1.68155200	-0.06060900
C	2.43877600	0.58406500	-0.22369000
H	3.39572500	0.09074600	-0.47982700
H	2.32178000	1.40783500	-0.93540800
H	2.50035000	0.98920100	0.78682600

C	-1.71969600	-1.14002200	-0.41380700
H	-2.26557700	-1.67655600	0.37324600
H	-2.38975100	-0.44127000	-0.91797600
H	-1.33539400	-1.86966300	-1.13429200
N	-0.60659500	-0.36197600	0.15388000

Path *b*

Optimized Coordinates at the B3LYP/6-31+G(d)/CPCM (methanol) level

16 atoms, **CO@aziridine [CH_3, CH_3] (Table 3)**

B3LYP/6-31+G(d). E = -325.8804994 H

C	-1.70440300	-1.99481500	-0.46475900
C	1.62481700	0.71998000	0.14124900
C	0.83314800	0.15765200	1.27527500
H	2.00959300	1.73084300	0.29823300
H	0.71367800	0.76945100	2.17031500
O	-2.68784000	-1.42851600	-0.37776600
C	-0.59172100	1.85866700	0.15605300
H	-0.13658500	2.58424400	0.85214600
H	-0.66124100	2.32533700	-0.83305100
H	-1.60588200	1.63548400	0.50647900
N	0.16544300	0.61229000	0.04840700
H	0.81745300	-0.91709500	1.44948400
C	2.45852100	-0.14268400	-0.77834100
H	2.51483900	0.29527400	-1.78316800
H	3.48180500	-0.23921800	-0.39417000
H	2.02912900	-1.14661800	-0.86600900

16 atoms, **P*b* [CH_3, CH_3]**

B3LYP/6-31+G(d). E = -325.9445216 H

C	-0.78538000	-0.61669900	0.33283300
C	1.12587100	0.25879600	0.31799700
C	0.57052500	-1.08836100	0.87606500
H	1.42270500	0.96091600	1.10739900
H	0.60564000	-1.20059500	1.96339100
O	-1.93336300	-1.05913900	0.32255500
C	-0.84107800	1.69028000	-0.79327200
H	-0.72930400	2.58205700	-0.16422100
H	-0.37133600	1.88106700	-1.76469800
H	-1.90544900	1.49498800	-0.94751600
N	-0.25305700	0.53478600	-0.15251900
H	0.96441200	-1.98853900	0.39458200
C	2.18440700	0.18770300	-0.77259200
H	2.34153200	1.16799500	-1.23798300
H	3.14005500	-0.13631100	-0.34398700
H	1.89555300	-0.52535000	-1.55420000

16 atoms, **TS*b* [CH_3, CH_3]**

B3LYP/6-31+G(d). E = -325.8004567 H

C	-0.87702000	-1.01074300	-0.16697500
C	1.17792500	0.34459700	0.15256500
C	0.82339700	-0.32074700	1.41122100
H	1.47713400	1.38515700	0.29060400
H	0.47149300	0.28174600	2.24137800
O	-2.10531500	-1.03003700	-0.24668000
C	-1.04189600	1.59312500	-0.16077400
H	-0.69961600	2.28898200	0.60953900
H	-0.87839700	2.04534500	-1.14569600
H	-2.10107200	1.37278000	-0.02224400

N	-0.29117700	0.32641100	-0.05363700
H	0.95684000	-1.38381500	1.56376200
C	2.05366700	-0.35324000	-0.87157800
H	1.95695000	0.13489300	-1.84715100
H	3.10021700	-0.28190300	-0.55440900
H	1.78076900	-1.40572000	-0.97363600

Optimized Coordinates at the B3LYP/6-31+G(d)/CPCM (dichloro-methane) level

16 atoms, **CO@aziridine [CH_3, CH_3] (Table 3)**

B3LYP/6-31+G (d). E = -325.8801704 H

C	-1.68118500	-1.98397200	-0.44916600
C	1.61661000	0.71626700	0.12860200
C	0.83883900	0.17466800	1.28242900
H	2.00426000	1.72938200	0.26346300
H	0.73161700	0.80228700	2.16814100
O	-2.66942300	-1.42358300	-0.37800900
C	-0.59959800	1.85569900	0.15010800
H	-0.13676500	2.59427400	0.82752500
H	-0.68100700	2.30404000	-0.84654700
H	-1.60954300	1.63861800	0.51621400
N	0.15660700	0.60810900	0.05638400
H	0.82432600	-0.89686600	1.47571500
C	2.43788200	-0.16301200	-0.78642000
H	2.48220100	0.25781400	-1.79914200
H	3.46571100	-0.25482500	-0.41328300
H	2.00552100	-1.16728600	-0.85208700

16 atoms, **P*b* [CH_3, CH_3]**

B3LYP/6-31+G(d). E = -325.943401 H

C	-0.78517700	-0.61587100	0.33731600
C	1.12612100	0.26167300	0.31436600
C	0.57268800	-1.08220200	0.88214200
H	1.42523200	0.96886900	1.09870200
H	0.60855900	-1.18686500	1.97022200
O	-1.93127400	-1.05877200	0.33039800
C	-0.84423500	1.68595700	-0.79959000
H	-0.73396800	2.58091700	-0.17446500
H	-0.37742200	1.87441200	-1.77305100
H	-1.90843200	1.48704500	-0.95065600
N	-0.25302000	0.53424000	-0.15558900
H	0.96689800	-1.98557000	0.40692300
C	2.18304300	0.18317700	-0.77764100
H	2.33908000	1.16000500	-1.25080900
H	3.13990100	-0.13745800	-0.34905100
H	1.89273900	-0.53548200	-1.55357700

16 atoms, **TS*b* [CH_3, CH_3]**

B3LYP/6-31+G(d). E = -325.7984625 H

C	-0.87760100	-1.01411000	-0.16185200
C	1.17808500	0.34614200	0.14919300
C	0.82916500	-0.31016100	1.41438500
H	1.47926200	1.38747700	0.27868200
H	0.48227800	0.29819000	2.24236500
O	-2.10346400	-1.03310800	-0.24121300
C	-1.04282400	1.59319100	-0.16794500
H	-0.70362000	2.29454100	0.59899400
H	-0.87895700	2.03997400	-1.15543300

H	-2.10191400	1.37117200	-0.03029000
N	-0.29125300	0.32785400	-0.05030600
H	0.95669000	-1.37335500	1.57101400
C	2.04888800	-0.35985000	-0.87391600
H	1.94825800	0.12097200	-1.85278200
H	3.09721800	-0.28806800	-0.56239400
H	1.77298600	-1.41229300	-0.96750100

Optimized Coordinates at the B3LYP/6-31+G(d)/CPCM (diethyl ether) level

16 atoms, **CO@aziridine [CH_3, CH_3] (Table 3)**

B3LYP/6-31+G (d). E = -325.8797219 H

C	-1.65757800	-1.97628500	-0.42147000
C	1.60832000	0.71283900	0.11087500
C	0.84813600	0.19837600	1.28885400
H	2.00045100	1.72771000	0.21817300
H	0.75742600	0.84540700	2.16250700
O	-2.65214500	-1.42411000	-0.37700400
C	-0.60565700	1.85633600	0.14173900
H	-0.13209500	2.61007800	0.79502900
H	-0.70196700	2.28161000	-0.86364100
H	-1.61018300	1.64879300	0.52772800
N	0.14772400	0.60653400	0.06509000
H	0.83407100	-0.86867700	1.50562500
C	2.41287800	-0.18765500	-0.79831300
H	2.44302800	0.21240700	-1.81989400
H	3.44592900	-0.27586100	-0.43889100
H	1.97551800	-1.19101800	-0.83749300

16 atoms, **P*b* [CH_3, CH_3]**

B3LYP/6-31+G(d). E = -325.9417952 H

C	-0.78484000	-0.61440300	0.34430000
C	1.12659800	0.26575700	0.30854200
C	0.57600300	-1.07297000	0.89101800
H	1.42915700	0.98053600	1.08514000
H	0.61342300	-1.16630400	1.98011100
O	-1.92836200	-1.05773500	0.34299200
C	-0.84887500	1.67941900	-0.80909300
H	-0.74006100	2.57915700	-0.19011100
H	-0.38689400	1.86391900	-1.78585200
H	-1.91296600	1.47559100	-0.95471500
N	-0.25296900	0.53328500	-0.16034400
H	0.97045100	-1.98106100	0.42505000
C	2.18095200	0.17607700	-0.78559100
H	2.33535800	1.14761200	-1.27030700
H	3.13961000	-0.13958000	-0.35712400
H	1.88838800	-0.55086200	-1.55294600

16 atoms, **TS*b* [CH_3, CH_3]**

B3LYP/6-31+G(d). E = -325.7956763 H

C	-0.87882300	-1.01900800	-0.15366800
C	1.17830700	0.34826500	0.14383100
C	0.83892600	-0.29409600	1.41904300
H	1.48252600	1.39064200	0.26007900
H	0.49962900	0.32302800	2.24364500
O	-2.10126600	-1.03752900	-0.23262900
C	-1.04384200	1.59345200	-0.17867500
H	-0.70883100	2.30306800	0.58286000

H	-0.87930800	2.03178000	-1.17006200
H	-2.10285200	1.36943900	-0.04207200
N	-0.29151700	0.32999200	-0.04521700
H	0.95773300	-1.35737200	1.58190700
C	2.04118200	-0.37019600	-0.87782500
H	1.93374500	0.09904300	-1.86166500
H	3.09229200	-0.29717400	-0.57555600
H	1.76131100	-1.42266400	-0.95782200

Optimized Coordinates at the B3LYP/6-31+G(d)/CPCM (toluene) level

16 atoms, **CO@aziridine [CH_3, CH_3] (Table 3)**

B3LYP/6-31+G (d). E = -325.879078 H

C	-1.60750300	-1.94839500	-0.39233600
C	1.59021300	0.70181200	0.08225700
C	0.86050100	0.23129900	1.29764700
H	1.98929400	1.71795300	0.14534000
H	0.79731600	0.90807800	2.15114400
O	-2.61391500	-1.41622400	-0.36667300
C	-0.61953300	1.85347400	0.13185000
H	-0.12866500	2.62934600	0.74586700
H	-0.74082900	2.24272000	-0.88533700
H	-1.61439600	1.66195700	0.54982200
N	0.12940400	0.60066900	0.08055900
H	0.84773200	-0.82776400	1.55084300
C	2.36628200	-0.23221900	-0.81780300
H	2.37175600	0.13468600	-1.85213800
H	3.40784800	-0.31531600	-0.48240800
H	1.92186200	-1.23300100	-0.81391900

16 atoms, **P*b* [CH_3, CH_3]**

B3LYP/6-31+G(d). E = -325.939593 H

C	-0.78436400	-0.61178300	0.35502400
C	1.12748000	0.27110800	0.29938300
C	0.58092700	-1.05976900	0.90324100
H	1.43510100	0.99666400	1.06461300
H	0.62159200	-1.13703300	1.99352200
O	-1.92465200	-1.05505900	0.36308800
C	-0.85528400	1.66968500	-0.82315800
H	-0.74698000	2.57653600	-0.21386800
H	-0.40111100	1.84721100	-1.80518600
H	-1.91951900	1.45979500	-0.95956200
N	-0.25289900	0.53161800	-0.16744000
H	0.97506100	-1.97450600	0.45011100
C	2.17784400	0.16533600	-0.79781600
H	2.32991300	1.12914600	-1.29874100
H	3.13907100	-0.14341700	-0.36980700
H	1.88187300	-0.57309500	-1.55282500

16 atoms, **TS*b* [CH_3, CH_3]**

B3LYP/6-31+G(d). E = -325.7919952 H

C	-0.82120160	-0.98149462	-0.14094868
C	1.23880340	0.39527438	0.13509032
C	0.91537840	-0.22605062	1.42518632
H	1.54769940	1.43885038	0.23130732
H	0.58777240	0.40389038	2.24477832
O	-2.03905160	-0.99912862	-0.21984468
C	-0.98439160	1.63859438	-0.19462168

H	-0.65561860	2.35984938	0.55920332
H	-0.81764060	2.06470838	-1.19128968
H	-2.04353060	1.41254938	-0.06021268
N	-0.23191660	0.37769538	-0.03779368
H	1.02128540	-1.28929762	1.59721432
C	2.08907340	-0.34177862	-0.88461468
H	1.97088140	0.11025038	-1.87538068
H	3.14429040	-0.26707262	-0.59670568
H	1.80359640	-1.39405662	-0.94411368

Table S9:

<u>Path *a*</u>

Optimized Coordinates at the B3LYP/6-31+G(d)/CPCM (methanol) level

13 atoms, **CO@aziridine [H, CH_3] (Table 4)**

B3LYP/6-31+G (d). E = -286.5708876 H

C	2.19596400	-1.00733900	0.95302400
C	-1.06771000	1.51654900	0.43912500
C	-1.71445500	0.53111300	-0.46577800
H	-1.38401100	2.55679700	0.39199300
H	-0.70557900	1.19203300	1.41283600
H	-2.46358400	0.93406600	-1.14821100
O	2.84519500	-1.09401700	0.02304600
C	-1.92690200	-0.91007400	-0.06554500
H	-2.89980300	-1.03502600	0.42634800
H	-1.90387300	-1.56867100	-0.94312900
H	-1.14586300	-1.23871100	0.62903500
N	-0.32035000	0.95968800	-0.71375700
H	-0.29083000	1.64348100	-1.47007500

13 atoms, **P*a* [H, CH_3]**

B3LYP/6-31+G(d). E = -286.6271837 H

C	0.74049500	-0.16527600	-0.10488900
C	-0.70901500	1.34610200	0.08194200
C	-0.76415800	-0.11965600	-0.45780300
H	-0.92933900	2.13247400	-0.64612700
H	-1.27200000	1.51300000	1.00624800
H	-0.91618500	-0.15601700	-1.54242800
O	1.62512400	-1.01381900	-0.14983200
C	-1.66869600	-1.11942500	0.25393100
H	-2.72219000	-0.90558000	0.04080300
H	-1.45582700	-2.13968100	-0.08546500
H	-1.52537800	-1.08394500	1.34062900
N	0.73222200	1.13041400	0.30671000
H	1.45670900	1.71728600	0.70773800

13 atoms, **TS*a* [H, CH_3]**

B3LYP/6-31+G(d). E = -286.4934874 H

C	1.28068600	-0.44579300	0.38319100
C	-0.61329800	1.20724400	0.33751500
C	-1.29937600	0.16606900	-0.43466400
H	-0.80079600	2.22284200	-0.01710500
H	-0.71343400	1.13871600	1.42059200
H	-1.33807600	0.33426500	-1.50851600
O	2.37558600	-0.71508300	-0.12323800
C	-1.98119900	-1.03164600	0.09197700
H	-3.05710400	-0.88038700	-0.12145100
H	-1.70324700	-1.94270400	-0.44869200
H	-1.84436500	-1.17026500	1.16460400
N	0.69992100	0.75551800	-0.13977500
H	1.23201000	1.35432800	-0.79321700

17 atoms, **CO@aziridine [OCH_3, CH_3] (Table 4)**

B3LYP/6-31+G (d). E = -401.0531349 H

C	0.03718900	2.78913700	-0.84925600
C	-0.66179100	-1.94167600	-0.57290200
C	-1.26552500	-1.06003900	0.46172400
H	-0.14605300	-2.83914200	-0.24285900

O	0.67958000	2.94108300	0.07725300
N	-0.01891000	-0.64728900	-0.20485100
H	-1.11606600	-1.38999300	1.48866800
H	-1.06467600	-1.96683000	-1.58301100
C	-2.49129000	-0.21253300	0.21682400
H	-3.39708900	-0.78506300	0.45029900
H	-2.48048100	0.68273500	0.84998600
H	-2.54877300	0.10494200	-0.82966900
O	1.08147000	-0.88357900	0.70987000
C	2.29974500	-0.83424400	-0.02785700
H	2.41746600	0.13551100	-0.52621800
H	3.09582600	-0.97393100	0.70812300
H	2.34379800	-1.63969900	-0.77345600

17 atoms, **P*a* [OCH_3, CH_3]**

B3LYP/6-31+G(d). E = -401.1002971 H

C	-0.30845100	0.83319500	0.23170800
C	-0.62546800	-1.26581800	0.37553900
C	-1.58520700	-0.03407900	0.33742200
H	-0.55060100	-1.75816500	1.34983700
O	-0.04018200	2.02546400	0.25163500
N	0.45970100	-0.29349900	0.13425600
H	-2.06573300	0.12880300	1.30854300
H	-0.74284400	-1.99883300	-0.42692100
C	-2.59172400	0.06322600	-0.80343300
H	-3.39357400	-0.67171400	-0.67208000
H	-3.04732900	1.05944700	-0.83018400
H	-2.11248400	-0.12115400	-1.77209200
O	1.81242400	-0.42942700	0.38689100
C	2.52701100	-0.85638800	-0.79470100
H	2.41303700	-0.11918300	-1.59552600
H	3.57111900	-0.92098100	-0.48221100
H	2.17301100	-1.83859600	-1.12444100

17 atoms, **TS*a* [OCH_3, CH_3]**

B3LYP/6-31+G(d). E = -400.9720581 H

C	-0.08793600	1.39122700	-0.38852100
C	-0.51671600	-1.10153200	-0.55272800
C	-1.57146100	-0.71340800	0.37886300
H	-0.03085100	-2.05001800	-0.32577300
O	0.61580800	2.29395400	0.02822000
N	0.29957900	0.01214200	0.01045800
H	-1.39802900	-1.00045900	1.41305900
H	-0.72420600	-0.99122800	-1.61579600
C	-2.88585600	-0.11699300	0.04392200
H	-3.63746000	-0.90479000	0.23828900
H	-3.14749200	0.71783700	0.70158200
H	-2.95652100	0.19399900	-0.99896400
O	1.49193300	-0.35732900	0.70540900
C	2.50170700	-0.87815700	-0.18291300
H	2.78879300	-0.12243300	-0.92004400
H	3.34960400	-1.11947600	0.46232200
H	2.15875000	-1.78824300	-0.68865400

18 atoms, **CO@aziridine [$COCH_3$, CH_3] (Table 4)**

B3LYP/6-31+G (d). E = -439.239614 H

C	2.07867100	3.12244900	-0.21453600
C	0.08193600	-2.43420200	0.39700700

C	0.89837100	-1.33677100	-0.23851800
H	-0.48769600	-3.09345100	-0.25548300
O	1.02399500	3.54072100	-0.28973600
N	-0.33197500	-1.03898200	0.47401600
C	-1.47685400	-0.46860400	-0.04555800
H	0.82283300	-1.26687500	-1.32428600
H	0.41820600	-2.87841600	1.33165900
C	2.20738800	-0.86859900	0.35222700
H	2.19859400	-0.95817800	1.44388900
H	3.03394500	-1.47301200	-0.03941900
H	2.40004400	0.17830900	0.09235300
O	-1.43392900	0.53205100	-0.76415700
C	-2.77915600	-1.11159400	0.38053100
H	-3.56814500	-0.35635800	0.41827700
H	-3.06562000	-1.86489300	-0.36529000
H	-2.69071900	-1.61242100	1.34854900

18 atoms, **P*a* [COCH$_3$, CH$_3$]**

B3LYP/6-31+G(d). E = -439.2932782 H

C	0.79382900	0.66801700	-0.22808100
C	0.46665300	-1.41714500	-0.14440100
C	1.72886500	-0.53751600	-0.39268700
H	0.16376800	-2.05985200	-0.97380000
O	0.92004000	1.87361300	-0.22889600
N	-0.32617100	-0.16189000	-0.05425300
C	-1.68678900	0.08088400	0.08856800
H	2.07558400	-0.60797700	-1.43019100
H	0.47529100	-1.97542100	0.79553500
C	2.90031500	-0.64652400	0.57887900
H	2.56413800	-0.58149500	1.62017400
H	3.41739200	-1.60265600	0.44375500
H	3.62177700	0.15784500	0.39897500
O	-2.13045200	1.22387500	0.12152200
C	-2.54734800	-1.15709000	0.18241700
H	-3.58288800	-0.86007500	0.35499200
H	-2.48709100	-1.73458000	-0.74796600
H	-2.21208800	-1.80912400	0.99698100

18 atoms, **TS*a* [COCH$_3$, CH$_3$]**

B3LYP/6-31+G(d). E = -439.1619614 H

C	1.39862200	1.69449500	-0.14344000
C	0.33728800	-1.10102200	0.74453900
C	1.34865900	-1.02377500	-0.30676700
H	-0.19273200	-2.05788200	0.56117200
O	1.05377300	2.78033400	-0.19463200
N	-0.52624600	0.07382200	0.66234800
C	-1.73681800	-0.00149100	0.08884000
H	0.97646600	-0.85458900	-1.31639400
H	0.78933800	-1.18606600	1.73883900
C	2.78399300	-1.24737400	-0.14584100
H	3.13103700	-1.15560600	0.88453300
H	2.93509700	-2.29868100	-0.46690000
H	3.38460100	-0.64448700	-0.83363400
O	-2.51593100	0.99121200	0.12480400
C	-2.22572900	-1.27203200	-0.61610900
H	-3.18338700	-1.06539200	-1.09839500
H	-1.52305300	-1.63047600	-1.37790000

H	-2.37247100	-2.08875200	0.10353100

26 atoms, **CO@aziridine [CH_3, CH_2Ph] (Table 4)**
B3LYP/6-31+G (d). E = -556.9405106 H

C	-3.90984900	1.26430000	1.78209000
C	-1.26135700	-1.83301300	-0.20158100
C	-0.78530400	-0.48303800	-0.62435000
H	-1.31806700	-2.61463400	-0.96010900
O	-4.53579400	1.69139100	0.93304100
N	-2.19743400	-0.70616200	-0.31371100
H	-0.54298600	-0.38145000	-1.68526200
H	-1.07193200	-2.18903700	0.80970100
C	0.00737000	0.43899500	0.29227100
H	-0.26536400	1.47809400	0.06490000
H	-0.29486700	0.24570700	1.32825500
C	1.50527800	0.25859200	0.13986800
C	2.23754000	1.04875200	-0.75997900
C	2.19022100	-0.72463100	0.87236200
C	3.61362300	0.86104100	-0.92876600
H	1.72645300	1.82157700	-1.33097900
C	3.56531800	-0.91727500	0.70725000
H	1.64289000	-1.34156400	1.58196800
C	4.28251400	-0.12490800	-0.19601400
H	4.16206300	1.48690600	-1.62861900
H	4.07664900	-1.68219600	1.28671300
H	5.35216800	-0.27096400	-0.32319600
C	-3.11692300	-0.74238200	-1.45062200
H	-4.00317300	-1.32664900	-1.17869300
H	-3.43449500	0.27848300	-1.68996400
H	-2.66979500	-1.18969100	-2.35481800

26 atoms, **P*a* [CH_3, CH_2Ph]**
B3LYP/6-31+G(d). E = -557.0015836 H

C	-2.51101000	0.56488300	0.06718100
C	-1.38359200	-1.00138600	-0.75050900
C	-1.01221400	0.43811800	-0.27493200
H	-1.24627000	-1.20181800	-1.81834400
O	-3.23079200	1.43433600	0.55502700
N	-2.77575400	-0.68792700	-0.38534600
H	-0.74163800	1.09327100	-1.11021600
H	-0.95471200	-1.81543400	-0.15558600
C	-0.01815800	0.59682200	0.88262000
H	-0.09830900	1.62549700	1.25813900
H	-0.31037100	-0.06530000	1.70691600
C	1.41500900	0.30791700	0.47584600
C	2.10170100	1.17638200	-0.38980900
C	2.08405100	-0.83401200	0.94067600
C	3.41416900	0.90702700	-0.78617300
H	1.60637300	2.07430300	-0.75438700
C	3.40031800	-1.10682900	0.54941100
H	1.57311700	-1.51587300	1.61746300
C	4.06911000	-0.23855800	-0.31819500
H	3.92711400	1.59297500	-1.45603200
H	3.90017800	-1.99690500	0.92369400
H	5.09087500	-0.44841700	-0.62405000
C	-3.99735000	-1.45098300	-0.51325000
H	-3.93660500	-2.38146900	0.06339900

H	-4.82447600	-0.84780800	-0.13018500
H	-4.19124700	-1.69738000	-1.56415600

26 atoms, **TS*a* [CH_3, CH_2Ph]**

B3LYP/6-31+G(d). E = -556.8645323 H

C	-3.05947759	0.51198737	0.81701775
C	-1.51442759	-1.26349363	0.08046875
C	-0.73723359	-0.05249863	-0.21061525
H	-1.40344859	-2.07678163	-0.63861325
O	-4.05597959	1.19121037	0.55670375
N	-2.73261759	-0.49189663	-0.19724325
H	-0.67571359	0.21978837	-1.26151425
H	-1.46499459	-1.63033963	1.10526475
C	0.11489841	0.72690537	0.73752375
H	-0.05209059	1.80246237	0.62762875
H	-0.08819059	0.44439137	1.77184275
C	1.55258541	0.38001137	0.34097175
C	2.23669041	1.14507937	-0.61721325
C	2.19373741	-0.73184563	0.91081275
C	3.54305741	0.81460537	-0.98645825
H	1.74975041	2.00822137	-1.06559525
C	3.50127241	-1.06125063	0.54341975
H	1.67380641	-1.33127063	1.65434275
C	4.17817041	-0.28987963	-0.40733425
H	4.06569141	1.42091637	-1.72160925
H	3.99051641	-1.91725263	1.00067375
H	5.19513141	-0.54542563	-0.69287625
C	-3.55736859	-0.82934863	-1.36780425
H	-4.08681959	-1.77662263	-1.20603125
H	-4.27641659	-0.02287363	-1.51836825
H	-2.92331559	-0.92980363	-2.25417625

16 atoms, **CO@aziridine [CH_3, CH_2Cl] (Table 4)**

B3LYP/6-31+G (d). E = -785.4780698 H

C	2.12518700	2.31144600	-0.79792300
C	0.18506600	-1.69650400	-0.84737700
C	-0.27605400	-0.88491200	0.31526400
H	0.33349300	-2.76400400	-0.68359800
O	2.77493800	2.33361800	0.13616200
N	1.09958000	-0.76926900	-0.17468800
H	-0.41993900	-1.41792100	1.25615400
H	-0.10811300	-1.42059800	-1.85867900
C	-1.12207800	0.34733500	0.14660100
H	-0.99090400	1.05132700	0.97002400
H	-0.92194100	0.84454000	-0.80241600
C	2.14088400	-1.36197200	0.66614200
H	3.00890900	-1.59818300	0.04137000
H	2.44560900	-0.63401300	1.42533000
H	1.81451100	-2.28427200	1.17451500
Cl	-2.90618900	-0.08174300	0.14129200

16 atoms, **P*a* [CH_3, CH_2Cl]**

B3LYP/6-31+G(d). E = -785.5383268 H

C	1.27848000	0.60708300	0.22185800
C	0.39820000	-1.29336700	0.08325100
C	-0.16447200	0.10282500	0.48586500
H	0.39205500	-2.04865700	0.87539400
O	1.84720900	1.69269400	0.27944000

N	1.70681600	-0.63959400	-0.09594500
H	-0.42472700	0.17274900	1.54657900
H	-0.00691100	-1.71250400	-0.84419400
C	-1.23703300	0.73267000	-0.37511500
H	-1.34746400	1.79573600	-0.15799000
H	-1.05066600	0.59340400	-1.44129200
C	3.01225500	-1.17508800	-0.41670200
H	3.00075200	-1.65796900	-1.40060600
H	3.73011700	-0.35119900	-0.43122100
H	3.32336800	-1.90851600	0.33666500
Cl	-2.87584600	-0.01852200	-0.04558200

16 atoms, **TS*a* [CH_3, CH_2Cl]**
B3LYP/6-31+G(d). E = -785.4014917 H

C	1.51586038	1.01931112	-0.39865281
C	0.48029338	-1.17023688	-0.79283881
C	-0.32631262	-0.46504888	0.20747119
H	0.60064138	-2.24229288	-0.64704981
O	2.39624938	1.68463012	0.12972419
N	1.57479538	-0.42255888	-0.14700981
H	-0.34134162	-0.87833488	1.21108419
H	0.32571638	-0.90803688	-1.83771081
C	-1.23230962	0.66240312	-0.08417081
H	-1.19844162	1.48157512	0.62810419
H	-1.21733262	1.00100512	-1.11397081
C	2.66979638	-1.13120188	0.53935219
H	3.35150638	-1.57141288	-0.19722881
H	3.20358338	-0.41085388	1.16061119
H	2.25462038	-1.92915488	1.15997419
Cl	-2.99412962	-0.07180388	0.17052519

Path *b*
Optimized Coordinates at the B3LYP/6-31+G(d)/CPCM (methanol) level
13 atoms, **CO@aziridine [H, CH_3] (Table 4)**
B3LYP/6-31+G (d). E = -286.5710182 H

C	2.19129000	-0.76520700	-1.05818100
C	-1.79600100	0.35903300	0.48728100
C	-0.85381700	1.34296100	-0.10622900
H	-2.59805300	0.77746500	1.09623200
H	-1.02233200	2.40313300	0.07249800
O	3.01439100	-0.52237400	-0.31170800
N	-0.42963700	0.49959800	1.03667500
H	-0.44880600	1.04627500	1.89758600
H	-0.36021600	1.11022800	-1.04781400
C	-2.12844700	-0.94802800	-0.19353000
H	-2.36965400	-1.72414200	0.54396900
H	-2.99517400	-0.82915100	-0.85585300
H	-1.28223900	-1.29874000	-0.79464300

13 atoms, **P*b* [H, CH_3]**
B3LYP/6-31+G(d). E = -286.6299643 H

C	0.99302500	0.14823400	-0.09255300
C	-1.06306200	0.18212400	0.38180100
C	-0.19826200	0.91412500	-0.69389000
H	-1.45223200	0.85899700	1.15029400
H	-0.13136400	2.00043200	-0.59048900
O	2.19909400	0.08977000	-0.30927200

N	0.19490400	-0.46903500	0.81771900
H	0.40454100	-1.11786200	1.57059200
H	-0.42650300	0.65007900	-1.73050200
C	-2.14557200	-0.76527800	-0.11132100
H	-2.52666400	-1.38467400	0.70914500
H	-2.98642000	-0.19339900	-0.52138500
H	-1.76001000	-1.42759000	-0.89545400

13 atoms, **TS*b* [H, CH_3]**
B3LYP/6-31+G(d). E = -286.4883984 H

C	1.23550700	-0.28786400	-0.42847100
C	-1.09317100	0.20694100	0.39967800
C	-0.64449800	1.34615300	-0.40397600
H	-1.54764400	0.51095100	1.34458900
H	-0.40052800	2.27938500	0.09104000
O	2.42596700	-0.32705000	-0.11065800
N	0.35606800	0.02221400	0.65925400
H	0.72464400	0.14703900	1.62100100
H	-0.54960600	1.28598200	-1.48132100
C	-1.83726800	-0.94451900	-0.24927100
H	-1.83583600	-1.81498400	0.41465400
H	-2.87648400	-0.64680600	-0.42675600
H	-1.37817800	-1.22493500	-1.20048000

17 atoms, **CO@aziridine [OCH_3, CH_3] (Table 4)**
B3LYP/6-31+G (d). E = -401.053111 H

C	0.59705700	2.61345400	-1.10650300
C	-1.45789000	-1.02573900	0.32859000
C	-1.14607000	0.13904900	1.19932900
H	-1.37798300	-1.99831500	0.81061500
O	1.67485500	2.61094400	-0.74195800
N	-0.15488100	-0.31376000	0.20817500
H	-0.89570700	-0.05346200	2.23931500
O	0.88495900	-1.03229200	0.92093900
C	1.67902600	-1.73401200	-0.03236200
H	2.47533000	-2.21329700	0.54307000
H	1.08938300	-2.50094900	-0.55211800
H	2.11202500	-1.04269100	-0.76554700
H	-1.57221500	1.11827400	0.99211600
C	-2.39267500	-0.93813600	-0.85382700
H	-2.12068900	-1.66908500	-1.62480800
H	-3.42315300	-1.14262100	-0.53830000
H	-2.36726200	0.06112600	-1.30136000

17 atoms, **P*b* [OCH_3, CH_3]**
B3LYP/6-31+G(d). E = -401.103178 H

C	0.17334200	1.25140800	0.06855200
C	-1.25846100	-0.28891300	0.39229900
C	-1.36233400	1.24920800	0.15492400
H	-1.38863600	-0.54309500	1.45081800
O	1.06286900	2.08736800	0.05377500
N	0.18668900	-0.12080600	0.07080700
H	-1.74676200	1.82178000	1.00263900
O	1.23689200	-0.93324700	0.46827900
C	1.71785900	-1.74800200	-0.62439300
H	2.54902500	-2.31715000	-0.20320800
H	0.93468900	-2.42858200	-0.97305300
H	2.06859400	-1.11549700	-1.44601700

H	-1.86989300	1.54494000	-0.76680500
C	-2.03448200	-1.23181800	-0.50888900
H	-1.72219000	-2.27086900	-0.35436900
H	-3.10443200	-1.16726600	-0.27909600
H	-1.88895000	-0.97531600	-1.56437800

17 atoms, **TS*b* [OCH$_3$, CH$_3$]**
B3LYP/6-31+G(d). E = -400.9689405 H

C	0.22459200	1.49776400	-0.45132400
C	-1.16753800	-0.50998300	0.27547900
C	-1.43455200	0.60046200	1.18496900
H	-1.01107500	-1.45312200	0.80030100
O	1.30103100	2.05642900	-0.51061500
N	0.18155100	0.15804300	0.19960700
H	-1.10362100	0.52663200	2.21454700
O	1.31572500	-0.59666700	0.63544700
C	1.57369400	-1.74766100	-0.19728400
H	2.47583700	-2.19877200	0.22182000
H	0.75106600	-2.46942900	-0.14895900
H	1.74990800	-1.43918600	-1.23189800
H	-1.99665200	1.47261200	0.87620900
C	-1.95978100	-0.67912500	-1.00629700
H	-1.41975700	-1.31666800	-1.71312700
H	-2.91481400	-1.16203100	-0.77008300
H	-2.15428700	0.28682800	-1.47797000

18 atoms, **CO@aziridine [COCH$_3$, CH$_3$] (Table 4)**
B3LYP/6-31+G (d). E = -439.2397223 H

C	-2.37117100	2.91092200	-0.27741200
C	-0.44247400	-1.86575700	0.33968600
C	-0.96088100	-0.46839400	0.56522600
H	0.07236700	-2.31155900	1.19142000
O	-1.42145900	3.45661200	0.02807900
N	0.21968100	-0.70234100	-0.24453800
C	1.49005200	-0.22480300	0.00834400
H	-0.80935800	-0.00662100	1.53925000
O	1.69008500	0.94166100	0.35269300
C	2.61586400	-1.20817800	-0.22982300
H	3.50003200	-0.67604000	-0.58948900
H	2.87493200	-1.68837000	0.72275000
H	2.33487900	-1.99089000	-0.93913800
H	-1.84767200	-0.14236000	0.02591800
C	-1.12901100	-2.83921300	-0.58814400
H	-1.88138900	-3.41593500	-0.03748900
H	-1.62724500	-2.31101700	-1.40809100
H	-0.40796900	-3.54459800	-1.01745100

18 atoms, **P*b* [COCH$_3$, CH$_3$]**
B3LYP/6-31+G(d). E = -439.294102 H

C	-0.97452100	0.95557600	0.21086300
C	-0.80646600	-1.14635200	0.45099000
C	-1.95651300	-0.11953600	0.66478500
H	-0.44235500	-1.58553900	1.38412700
O	-1.00468200	2.15960700	0.07446200
N	0.05233700	0.01352400	0.03157000
C	1.41603900	0.16076800	-0.20492700
H	-2.28920800	0.00382700	1.69893100

O	1.88064300	1.23590800	-0.56845600
C	2.26033600	-1.06655600	0.04736400
H	3.30087700	-0.83320100	-0.18550600
H	2.18208400	-1.37329300	1.09843600
H	1.93233900	-1.91093800	-0.56909500
H	-2.82288600	-0.24278600	0.00873200
C	-1.04341300	-2.20377300	-0.61642400
H	-1.82397900	-2.89141400	-0.27095700
H	-1.37713900	-1.74585000	-1.55453000
H	-0.14458300	-2.79536500	-0.81687400

18 atoms, **TS*b* [COCH$_3$, CH$_3$]**

B3LYP/6-31+G(d). E = -439.1494234 H

C	-1.54911800	1.52890700	0.19982400
C	-0.56676300	-1.25925500	0.08807500
C	-1.25277000	-0.60235000	1.19385700
H	0.14295100	-1.98897400	0.50652600
O	-1.23936100	2.60951300	-0.00494700
N	0.14131800	-0.07956800	-0.46853000
C	1.45193300	0.13884700	-0.21732300
H	-0.67101300	-0.22448800	2.02986000
O	2.04552500	1.05303700	-0.84134600
C	2.23686500	-0.69328900	0.79496200
H	3.22032300	-0.24114600	0.93845200
H	1.73713000	-0.76114800	1.76794700
H	2.37787200	-1.71773700	0.42614000
H	-2.33199700	-0.56401900	1.29340200
C	-1.46375300	-1.93512600	-0.94366300
H	-2.03692100	-2.74546800	-0.47868300
H	-2.16108400	-1.21141500	-1.37845700
H	-0.85417100	-2.35543600	-1.74953900

26 atoms, **CO@aziridine [CH$_3$, CH$_2$Ph] (Table 4)**

B3LYP/6-31+G (d). E = -556.940663 H

C	3.86029600	1.81294700	-1.12048200
C	0.73041200	-0.72361800	0.44189000
C	1.42588100	0.24553200	1.33873700
H	0.38962700	-1.64608100	0.91864600
O	4.88334000	1.36782200	-0.89561500
N	2.17981300	-0.54781600	0.35928800
H	1.53217500	-0.01843800	2.39157300
C	3.00294900	-1.62975000	0.89886900
H	3.14259700	-2.39513400	0.12734100
H	3.98576800	-1.23117900	1.17449000
H	2.56029900	-2.10847000	1.78910400
H	1.37089500	1.31394300	1.13671900
C	-0.10221500	-0.28787600	-0.75645600
H	0.02101700	-1.03306900	-1.55412400
H	0.29750900	0.65925600	-1.13610600
C	-1.57291200	-0.13903900	-0.41930500
C	-2.42456900	-1.25559800	-0.42791500
C	-2.11277200	1.10708900	-0.06399100
C	-3.77504000	-1.13402900	-0.08637100
H	-2.02728800	-2.22890300	-0.70991100
C	-3.46358300	1.23500200	0.27696500
H	-1.47148200	1.98607000	-0.05922600

C	-4.29973700	0.11362000	0.26841400
H	-4.41732200	-2.01122700	-0.10261300
H	-3.86244900	2.21036300	0.54508900
H	-5.35018500	0.21152000	0.53039900

26 atoms, **P*b* [CH$_3$, CH$_2$Ph]**

B3LYP/6-31+G(d). E = -557.0031095 H

C	2.91216600	0.73768700	0.23794400
C	1.02567400	-0.18530000	0.31424300
C	1.51505100	1.27454500	0.57904700
H	0.69649800	-0.70318400	1.22396500
O	4.05018400	1.20066900	0.20451700
N	2.43278800	-0.50962700	-0.01214900
H	1.41009600	1.62910500	1.60784600
C	3.09035300	-1.76971500	-0.28392300
H	2.70074000	-2.22767000	-1.19949100
H	4.15892200	-1.57918800	-0.41218400
H	2.94929200	-2.46797900	0.55036200
H	1.14037700	2.03128500	-0.11548400
C	0.03332300	-0.42906200	-0.83247500
H	0.15879900	-1.46104900	-1.18225200
H	0.29817300	0.22619000	-1.67264200
C	-1.41405100	-0.22256100	-0.42752600
C	-2.24576600	-1.32617000	-0.18016000
C	-1.95638500	1.06487400	-0.27907400
C	-3.57939400	-1.15336400	0.20518800
H	-1.84725400	-2.33191800	-0.29722300
C	-3.28691400	1.24326800	0.11231600
H	-1.33858400	1.93731700	-0.47904400
C	-4.10439400	0.13387300	0.35542100
H	-4.20621700	-2.02346800	0.38458100
H	-3.68639200	2.24873800	0.21990800
H	-5.14050200	0.27203500	0.65361200

26 atoms, **TS*b* [CH$_3$, CH$_2$Ph]**

B3LYP/6-31+G(d). E = -556.860119 H

C	2.96026606	0.87690356	-0.45128216
C	0.94796106	-0.33352144	0.34395284
C	1.27716506	0.73062856	1.29873584
H	0.66710106	-1.27059144	0.82789784
O	4.18587406	0.89616256	-0.55101716
N	2.41098006	-0.35332744	0.12968084
H	1.64335206	0.45550456	2.28170684
C	3.19251606	-1.56607544	0.44412184
H	3.03168006	-2.32849644	-0.32663616
H	4.24675306	-1.28913644	0.48124384
H	2.87545906	-1.96613544	1.41066384
H	1.12755906	1.77942456	1.07804284
C	0.04906506	-0.04810744	-0.86299716
H	0.23173206	-0.83224144	-1.60724916
H	0.34765306	0.90489556	-1.30761316
C	-1.41490594	-0.02595644	-0.46925016
C	-2.15867594	-1.21552044	-0.40544316
C	-2.05125294	1.18002456	-0.13642116
C	-3.50072994	-1.20191944	-0.01494916
H	-1.68543994	-2.15938144	-0.66923916
C	-3.39425794	1.19813956	0.25549284

H	-1.49643894	2.11395056	-0.19375516
C	-4.12285494	0.00645056	0.31872084
H	-4.06090194	-2.13278344	0.02423284
H	-3.87019494	2.14290056	0.50587484
H	-5.16719094	0.01887056	0.61974484

16 atoms, **CO@aziridine [CH_3, CH_2Cl] (Table 4)**
B3LYP/6-31+G (d). E = -785.4782231 H

C	-2.44075000	-2.12859000	-0.47436400
C	0.47872600	0.90830400	0.20064000
C	-0.25856900	0.39436400	1.39019000
H	0.81573000	1.94493700	0.24299300
O	-3.49189100	-1.69344100	-0.50766500
N	-0.96709200	0.68402600	0.14058100
H	-0.40159600	1.08121500	2.22453600
C	-1.81348200	1.87802900	0.12254900
H	-1.90774800	2.23504500	-0.90842900
H	-2.80957300	1.61124400	0.49167300
H	-1.41858900	2.69845100	0.74434000
H	-0.19255200	-0.65739300	1.66278700
C	1.29898900	0.00597700	-0.68037800
H	1.36686300	0.38190000	-1.70270400
H	0.90990300	-1.01210600	-0.68386700
Cl	3.02792000	-0.10105900	-0.07548800

16 atoms, **P*b* [CH_3, CH_2Cl]**
B3LYP/6-31+G(d). E = -785.5397822 H

C	-1.71752800	-0.63992700	0.35432500
C	0.12277000	0.36538900	0.33746600
C	-0.35182000	-0.97678000	0.97026900
H	0.37311200	1.14394900	1.06683700
O	-2.83155500	-1.15257900	0.34749800
N	-1.24537600	0.51287800	-0.20049800
H	-0.34857300	-1.00273600	2.06293800
C	-1.90245400	1.60472700	-0.88981100
H	-1.46055600	1.77291800	-1.87763200
H	-2.95598600	1.34153700	-1.01454600
H	-1.83100600	2.52841200	-0.30339300
H	0.12262700	-1.87867300	0.57332000
C	1.18066900	0.28673700	-0.75316300
H	1.27018600	1.22553500	-1.30181500
H	0.98192200	-0.53026600	-1.44928500
Cl	2.82078400	-0.03056000	-0.02127100

16 atoms, **TS*b* [CH_3, CH_2Cl]**
B3LYP/6-31+G(d). E = -785.3945697 H

C	-1.66298300	-1.05650200	-0.14286100
C	0.25854200	0.44711900	0.26592100
C	-0.12342800	-0.15773200	1.54424700
H	0.52258700	1.50290800	0.33759100
O	-2.86914500	-1.16611200	-0.33065500
N	-1.18019100	0.33455500	-0.04049200
H	-0.55046300	0.47859800	2.31079400
C	-1.98612700	1.54363000	-0.30770200
H	-1.76568200	1.92989100	-1.30915400
H	-3.03881500	1.26814800	-0.23640100
H	-1.74901500	2.31270800	0.43139600

H	0.04125200	-1.20421400	1.76510500
C	1.19327900	-0.29161100	-0.67695400
H	1.12451200	0.11260800	-1.68723300
H	1.00052200	-1.36209700	-0.68311000
Cl	2.91493600	-0.05652700	-0.12330700

Table S10:
Optimized Coordinates at the B3LYP/6-31+G(d) level in the gas phase
<u>Path *a'*</u>
18 atoms, **CO@Aziridine [CH_3, CH_3]** @NaBr
B3LYP /6-31+G(d). E = -3059.988427 H

C	3.26072200	-1.19826500	0.81186800
C	0.10620000	1.75189500	0.49071800
C	1.34181600	1.79132400	1.30033900
H	1.41205900	1.19893200	2.21117700
O	2.70155300	-2.00144800	0.22352100
Br	-1.99508900	-0.75276100	-0.04125000
Na	0.47626300	-1.10813400	-0.69975700
N	1.34772600	1.02848400	0.02839500
C	2.20103600	1.63119400	-1.00548300
H	3.25049700	1.54667100	-0.69168000
H	2.08632100	1.08914600	-1.95326900
H	1.99259300	2.69016800	-1.20353400
H	1.94350900	2.70094600	1.30695900
H	-0.63239200	1.02864900	0.81802500
C	-0.53462900	2.90112400	-0.25003200
H	-1.01526300	3.57288000	0.47194300
H	0.15943000	3.50155200	-0.84416300
H	-1.31776800	2.51038200	-0.90886600

18 atoms, **TS1*a'* [CH_3, CH_3]**
B3LYP/6-31+G(d). E = -3059.923444 H

C	3.03212400	-1.21012200	0.74732800
C	-0.50639100	1.63268600	0.75334800
C	0.90448200	1.29366700	1.04123300
H	0.96133000	0.62999600	1.91601900
O	2.58943400	-2.13916400	0.22880600
Br	-2.00553600	-0.73437800	0.01099800
Na	0.44125400	-1.28731300	-0.79243300
N	1.43792300	0.63447500	-0.14133000
C	2.31117600	1.48862100	-0.90810700
H	3.06476700	2.00388300	-0.27625700
H	2.84656100	0.89465500	-1.66240000
H	1.78820800	2.29199700	-1.46738800
H	1.46492100	2.21475600	1.31593000
H	-1.27164600	1.34604700	1.46579300
C	-0.93245300	2.56682500	-0.31679100
H	-0.97542900	3.57628900	0.13535900
H	-0.23732900	2.61562500	-1.15753500
H	-1.93598500	2.33234100	-0.68087300

18 atoms, **Int*a'* [CH_3, CH_3]**
B3LYP/6-31+G(d). E = -3059.952774 H

C	-0.99132500	-1.03890100	-0.87400000
C	0.20074900	-1.49011800	-0.00460900
N	-1.98922400	-0.20239600	-0.23613800
H	-0.61004500	-0.59400400	-1.80426700
H	-1.49117300	-1.97363500	-1.16413000
C	-3.37856700	-0.66193400	-0.26430100
H	-3.97968700	0.10564200	0.22851200
H	-3.49672400	-1.61501200	0.27034500
Br	1.83162600	-0.27086700	-0.22630400
Na	0.36312700	2.22657900	0.27763000

C	-0.06964100	-1.63629500	1.47832100
H	0.80256700	-2.03511000	2.00447300
H	-0.36059700	-0.67995000	1.91873900
H	-0.90901500	-2.33196300	1.61529400
H	0.62167800	-2.40596200	-0.42472200
C	-1.65466900	1.12450100	0.09218100
O	-2.56493500	1.86804100	0.51693000
H	-3.74834400	-0.79222700	-1.29347400

18 atoms, **TS2*a′* [CH_3, CH_3]**

B3LYP/6-31+G(d). E = -3059.938018 H

C	-1.18215200	-1.38756500	-0.57855200
C	0.06168000	-1.46261500	0.25644900
N	-2.06523900	-0.30554600	-0.22697700
H	-0.91470200	-1.37111200	-1.64642100
H	-1.70318500	-2.35251100	-0.40863300
C	-3.43950900	-0.30772200	-0.72516700
H	-3.88579300	0.65287600	-0.45656400
H	-4.02277300	-1.11915500	-0.27207400
Br	2.10386200	-0.02051300	-0.38916600
Na	0.52140100	2.02153000	0.45807100
C	-0.00022500	-1.51861500	1.75418200
H	0.95408500	-1.22509500	2.19822800
H	-0.79473100	-0.89485100	2.16427600
H	-0.17234600	-2.56978900	2.05026900
H	0.75431000	-2.18718600	-0.16043400
C	-1.40507200	0.81242000	0.22706400
O	-1.98327100	1.91163800	0.36462300
H	-3.47093000	-0.42174000	-1.81861100

18 atoms, **P*a′* [CH_3, CH_3]**

B3LYP/6-31+G(d). E = -3060.06338 H

C	-2.25440700	-1.95412200	-0.18442800
C	-0.97379100	-1.29878500	0.42527300
N	-2.81365100	-0.59310200	-0.29672900
H	-2.11993400	-2.44944900	-1.15248100
H	-2.82330000	-2.59878600	0.49574800
C	-4.04009500	-0.08644800	-0.86968500
H	-4.04201400	1.00167200	-0.76511600
H	-4.91296200	-0.49759700	-0.34856600
Br	2.33334800	0.19825700	-0.40396100
Na	0.59426900	1.93630900	0.39111300
C	-0.57209300	-1.66375000	1.84985200
H	0.23750500	-1.00971400	2.18970100
H	-1.41485400	-1.57629500	2.54727800
H	-0.19755100	-2.69295500	1.88790900
H	-0.10168100	-1.34519700	-0.23689900
C	-1.73000900	0.02196900	0.22900100
O	-1.49832100	1.21986100	0.43426700
H	-4.10804900	-0.34462500	-1.93391800

Path *b′*

18 atoms, **CO@Aziridine [CH_3, CH_3] @NaBr**

B3LYP /6-31+G(d). E = - 3060.002629 H

C	4.45515100	0.13673100	-0.28432400
C	-1.33197200	-1.69537300	0.78496700
C	-0.97123400	-2.17761000	-0.57351200
H	-1.70682000	-0.68149900	0.91451300

H	-1.05943600	-1.44205900	-1.37360100
O	3.32588100	0.24041300	-0.16956500
C	-1.20188400	-3.60169100	-1.03240900
H	-2.16841900	-3.65619000	-1.54709500
H	-0.43067400	-3.92288700	-1.74417400
H	-1.23108800	-4.31968000	-0.20781300
H	-1.68704100	-2.43389300	1.50432100
Br	-1.14993100	2.02979200	-0.05626100
Na	0.84363300	0.42160500	0.05576500
N	0.09616900	-1.80309800	0.39539700
C	0.87359000	-2.86976700	1.03467600
H	1.67533700	-3.19487000	0.36147900
H	1.32815500	-2.47067500	1.94877300
H	0.27970700	-3.74999700	1.31555300

18 atoms, **TS1*b′* [CH_3, CH_3]**
B3LYP/6-31+G(d). E = -3059.917781 H

C	4.71099200	0.26245800	-0.31985400
C	-1.85946100	-0.79278200	0.25862700
C	-0.92965100	-1.61384700	-0.54457200
H	-2.74093700	-0.32405800	-0.15932100
H	-0.88072000	-1.15273500	-1.54324500
O	3.62342400	0.58626400	-0.21238800
C	-1.41022400	-3.06575900	-0.77769200
H	-2.34830300	-3.07062300	-1.34632500
H	-0.65145500	-3.60521600	-1.36035500
H	-1.57867200	-3.60649400	0.15916800
H	-1.80138300	-0.83732700	1.34087400
Br	-1.23471200	1.80331000	0.03783600
Na	1.15508600	0.68346100	-0.05030400
N	0.36417700	-1.39450100	0.09623800
C	0.62939600	-2.32380400	1.16672600
H	0.75243500	-3.37296100	0.83618700
H	1.55194900	-2.04013400	1.69110400
H	-0.17686600	-2.35055300	1.93703500

18 atoms, **Int*b′* [CH_3, CH_3]**
B3LYP/6-31+G(d). E = -3059.937264 H

C	2.26435300	-0.82740000	1.14141200
C	-0.38166900	0.91664400	1.09814300
C	0.76014500	1.42482400	0.22656200
H	-0.05325300	0.20773000	1.85707600
H	1.41651000	1.90546300	0.99395900
O	2.01997400	-1.95173600	0.85412000
C	0.28694200	2.54790000	-0.71809600
H	-0.25526500	3.32553900	-0.16357200
H	1.13729800	3.02836300	-1.21138900
H	-0.37587600	2.14845300	-1.49459400
H	-0.95313500	1.72492900	1.55455900
Br	-1.79309500	-0.11812800	0.06365100
Na	0.47696500	-1.62239200	-0.83962000
N	1.46728000	0.33319900	-0.40735600
C	2.69806400	0.77717300	-1.04309400
H	3.34360300	1.36789800	-0.36179600
H	3.28489500	-0.09802100	-1.35827400
H	2.53363700	1.38824800	-1.94625500

18 atoms, **TS2*b′* [CH_3, CH_3]**

B3LYP/6-31+G(d). E = -3059.918321 H

C	1.99513503	-0.58546679	0.94588254
C	-0.01211997	0.99536921	1.53049454
C	0.97010603	1.51599221	0.54276354
H	-0.11121297	-0.03625979	1.79436354
H	1.66882403	2.13112021	1.14188154
O	2.40807903	-1.69124079	0.55986454
C	0.37198103	2.47906421	-0.50249246
H	-0.18167097	3.26999121	0.01398654
H	1.16388803	2.95311121	-1.09071246
H	-0.32301997	1.95326821	-1.15983146
H	-0.46457797	1.71945521	2.20777054
Br	-1.87803597	-0.14071579	-0.13150746
Na	0.27272203	-1.76565479	-0.50299646
N	1.69492603	0.38875121	-0.02801246
C	2.48783303	0.55037521	-1.25194546
H	3.21026403	1.37271721	-1.15518546
H	3.04241803	-0.37818079	-1.41198046
H	1.85319003	0.74790021	-2.12337446

18 atoms, **P*b'* [CH_3, CH_3]**

B3LYP/6-31+G(d). E = -3060.065404 H

C	1.66866500	-0.13991400	0.69108600
C	1.15997600	0.85671500	1.73171400
C	1.61696900	1.95402200	0.71622100
H	0.07951500	0.80015800	1.88978300
H	2.45607500	2.56403700	1.07538800
O	1.68453400	-1.37302800	0.57466100
C	0.52849500	2.81336700	0.08911200
H	0.15763000	3.53628100	0.82555900
H	0.91553200	3.38117900	-0.76621000
H	-0.31181100	2.19364800	-0.24361800
H	1.69572100	0.83496000	2.68507800
Br	-1.97600700	-0.36642300	-0.35346200
Na	-0.24051500	-2.25916800	-0.02500300
N	2.09918600	0.83411300	-0.13564200
C	2.66106100	0.79709800	-1.46633200
H	3.60560700	1.35315800	-1.50082100
H	2.85439100	-0.24654400	-1.72774000
H	1.96576400	1.22599100	-2.19746600

Table S11:

Optimized Coordinates at the PBE/6-31+G(d) level in the gas phase

48 atoms, **CO@Aziridine [CH_3, CH_3]** @ **$Mg_{16}O_{16}$**

PBE/6-31+G(d). E = - 4729.091162 H

Mg	3.36148300	-1.02337400	-1.64285500
Mg	1.70459200	0.78266200	-2.81912300
Mg	1.00740700	-1.96429300	-2.62521100
Mg	1.21849700	-0.36951400	-0.05517200
O	0.08136000	-0.22716700	-2.16582800
O	2.04086600	-2.17804300	-0.75156800
O	2.78118500	0.72680300	-0.95054200
O	2.55283400	-0.94040500	-3.39714400
Mg	1.81917400	2.41835400	-0.45672800
Mg	0.01407400	4.12659100	-1.55800500
Mg	-0.73849300	1.49354500	-1.53273900
Mg	-0.59872800	3.13183800	0.90294400
O	-1.65818800	3.29527300	-0.94294900
O	0.29480200	1.37810400	0.40020000
O	0.96389200	4.13352800	0.13738600
O	0.88518600	2.55238300	-2.35431800
Mg	0.41163100	-3.13119700	-0.06392200
Mg	-1.43630500	-1.25327400	-1.33903000
Mg	-2.02638200	-3.90305100	-0.99067400
Mg	-2.01190000	-2.42205500	1.29469800
O	-3.08280100	-2.30417600	-0.54809700
O	-1.13845200	-4.13602200	0.72148000
O	-0.41276900	-1.38768500	0.58925400
O	-0.53969100	-3.05284800	-1.95949300
Mg	-1.27651300	0.35100300	1.14715200
Mg	-3.13870500	2.21599900	-0.12273500
Mg	-3.84301000	-0.55290100	0.07242600
Mg	-3.65706600	1.03702500	2.28448900
O	-4.67971500	1.18152900	0.64054300
O	-2.87094600	-0.73771500	1.96455600
O	-2.13190000	2.16455100	1.76034000
O	-2.31999900	0.47958700	-0.79459900
C	1.56020800	-0.36197900	2.44613200
O	0.98559200	-0.20328400	3.44167600
C	4.72463100	-1.51044000	2.32540800
C	4.77472400	-0.00887100	2.26807300
H	4.20401900	-2.09017700	1.54992900
H	4.22704200	0.46617400	1.43816300
N	3.85966500	-0.70480800	3.18920300
C	4.07364100	-0.70562300	4.63197800
H	3.50129800	-1.54461800	5.06544400
H	5.13580800	-0.81798700	4.93713000
H	3.68088100	0.23438900	5.05703000
H	5.57506100	-2.02208200	2.80149600
C	5.97971200	0.77296800	2.75125700
H	6.65490900	0.97832500	1.90152900
H	5.67698300	1.74744900	3.17610100
H	6.56292100	0.23115400	3.51372600

48 atoms, **P_{2c} [CH_3, CH_3]**

PBE/6-31+G(d). E = -4729.156639 H

Mg	3.38746921	-1.03800057	-1.77491776

Mg	1.69218621	0.77874043	-2.88338876
Mg	1.00133921	-1.97172157	-2.66268176
Mg	1.29295121	-0.38499757	-0.09333576
O	0.14012021	-0.23917557	-2.13488076
O	2.13482121	-2.20708357	-0.81801276
O	2.89605921	0.71518143	-1.00977176
O	2.52610021	-0.94039057	-3.49245176
Mg	1.88304521	2.40279443	-0.53337676
Mg	0.06071321	4.11360543	-1.54758676
Mg	-0.71524479	1.46944143	-1.48296376
Mg	-0.45889279	3.11662743	0.92010424
O	-1.58064079	3.30333443	-0.85221776
O	0.38723221	1.37301643	0.34065124
O	1.09152621	4.12431643	0.10729324
O	0.90785021	2.54355143	-2.38782076
Mg	0.49214421	-3.13414957	-0.09452276
Mg	-1.39946679	-1.23148557	-1.27466576
Mg	-1.96771079	-3.90062857	-0.92076976
Mg	-1.86741979	-2.43198057	1.35416524
O	-3.00752979	-2.31932757	-0.41041776
O	-1.00704479	-4.15627957	0.75416624
O	-0.32130179	-1.40163657	0.56192824
O	-0.51761579	-3.05592857	-1.95150176
Mg	-0.89829679	0.30938843	1.52716324
Mg	-3.00397079	2.16560143	-0.00278576
Mg	-3.68783479	-0.53404957	0.20922924
Mg	-3.42027679	1.01761143	2.43408224
O	-4.51964179	1.17298943	0.85901224
O	-2.63001279	-0.74578057	2.10665724
O	-1.90287379	2.12847543	1.87873724
O	-2.35810179	0.48655643	-0.90489076
C	1.76332621	-0.37530557	2.29972424
O	0.80108221	0.01349843	2.99096424
C	4.02914321	-1.28816957	2.21500624
C	4.21511121	0.13249043	1.84374724
H	3.68482321	-1.95732257	1.40986224
H	3.74941221	0.47755543	0.89769224
N	2.91253821	-0.81889857	3.06005524
C	2.94288621	-0.96893157	4.52532824
H	2.82278721	-2.03017257	4.80972424
H	3.90579221	-0.60233557	4.91812824
H	2.11418421	-0.37515457	4.93774424
H	4.82098721	-1.73862057	2.83270124
C	5.03496521	1.08487543	2.63347024
H	5.95241821	1.30082543	2.04434524
H	4.52719521	2.05733043	2.76299924
H	5.35644621	0.69946443	3.61507024

48 atoms, **TS_{2c} [CH_3, CH_3]**

PBE/6-31+G(d). E = -4729.043898 H

Mg	3.33869000	-1.03514800	-1.81218200
Mg	1.64340700	0.78159300	-2.92065300
Mg	0.95256000	-1.96886900	-2.69994600
Mg	1.24417200	-0.38214500	-0.13060000
O	0.09134100	-0.23632300	-2.17214500
O	2.08604200	-2.20423100	-0.85527700

O	2.84728000	0.71803400	-1.04703600
O	2.47732100	-0.93753800	-3.52971600
Mg	1.83426600	2.40564700	-0.57064100
Mg	0.01193400	4.11645800	-1.58485100
Mg	-0.76402400	1.47229400	-1.52022800
Mg	-0.50767200	3.11948000	0.88284000
O	-1.62942000	3.30618700	-0.88948200
O	0.33845300	1.37586900	0.30338700
O	1.04274700	4.12716900	0.07002900
O	0.85907100	2.54640400	-2.42508500
Mg	0.44336500	-3.13129700	-0.13178700
Mg	-1.44824600	-1.22863300	-1.31193000
Mg	-2.01649000	-3.89777600	-0.95803400
Mg	-1.91619900	-2.42912800	1.31690100
O	-3.05630900	-2.31647500	-0.44768200
O	-1.05582400	-4.15342700	0.71690200
O	-0.37008100	-1.39878400	0.52466400
O	-0.56639500	-3.05307600	-1.98876600
Mg	-0.94707600	0.31224100	1.48989900
Mg	-3.05275000	2.16845400	-0.04005000
Mg	-3.73661400	-0.53119700	0.17196500
Mg	-3.46905600	1.02046400	2.39681800
O	-4.56842100	1.17584200	0.82174800
O	-2.67879200	-0.74292800	2.06939300
O	-1.95165300	2.13132800	1.84147300
O	-2.40688100	0.48940900	-0.94215500
C	1.71454700	-0.37245300	2.26246000
O	0.75230300	0.01635100	2.95370000
C	3.98036400	-1.28531700	2.17774200
C	4.16633200	0.13534300	1.80648300
H	3.63604400	-1.95447000	1.37259800
H	3.70063300	0.48040800	0.86042800
N	2.86375900	-0.81604600	3.02279100
C	2.89410700	-0.96607900	4.48806400
H	2.77400800	-2.02732000	4.77246000
H	3.85701300	-0.59948300	4.88086400
H	2.06540500	-0.37230200	4.90048000
H	4.77220800	-1.73576800	2.79543700
C	4.98618600	1.08772800	2.59620600
H	5.90363900	1.30367800	2.00708100
H	4.47841600	2.06018300	2.72573500
H	5.30766700	0.70231700	3.57780600

Table S12:

Optimized Coordinates at the PBE/6-31+G(d) level in the gas phase

48 atoms, **CO@Aziridine [CH_3, CH_3]** @ **$Mg_{16}O_{16}$**

PBE/6-31+G(d). E = - 4729.08865 H

Mg	-3.50986700	-1.03571000	2.46389800
Mg	-3.25388400	-1.96809600	-0.08277400
Mg	-3.76179000	0.79442900	0.46274300
Mg	-1.16834000	-0.35863400	1.21923900
O	-2.37100400	-0.21888700	-0.62367400
O	-2.63089500	0.71378000	2.27921300
O	-2.09973200	-2.18020700	1.70775100
O	-4.66420800	-0.94137200	0.90642800
Mg	-0.70137900	-3.12258200	0.62115400
Mg	-0.36128700	-3.88351800	-1.97460300
Mg	-0.93261600	-1.23065300	-1.61179400
Mg	1.63663800	-2.40327500	-0.87339700
O	0.52887400	-2.27989100	-2.67935900
O	0.28913600	-1.38001900	0.26541000
O	0.72166400	-4.12363300	-0.37814500
O	-1.91563000	-3.02855000	-1.12706900
Mg	-1.71824600	2.41135600	1.71310700
Mg	-1.43709600	1.51459000	-1.06813800
Mg	-1.84100700	4.13596300	-0.39677800
Mg	0.61570300	3.14272000	0.22039000
O	-0.50815500	3.31316300	-1.58183500
O	-0.80115600	4.13205400	1.24395700
O	-0.22382500	1.37428900	0.80107900
O	-2.94555800	2.55182000	-0.02576300
Mg	1.24056600	0.36329100	-0.16996700
Mg	1.44144600	-0.51546900	-3.03404400
Mg	0.93073500	2.23395600	-2.49661000
Mg	3.31090500	1.06950200	-1.81770600
O	2.34280200	1.21340000	-3.49722500
O	2.09051700	2.21063100	-0.75248300
O	2.64062200	-0.73102900	-1.31538000
O	-0.05510500	0.49994200	-2.11225000
C	3.54481700	0.61270500	0.96255800
O	4.59853800	0.90683900	0.54186500
C	2.75477200	-0.15573300	3.94870100
C	3.06038100	-1.42310800	3.20558900
H	1.95421700	0.51295600	3.60428800
H	2.47369200	-1.59526100	2.28919100
N	3.92381800	-0.23776900	3.07203100
C	5.27190800	-0.18156900	3.62233500
H	5.56171100	0.87873400	3.71935000
H	5.37645200	-0.66169600	4.61747400
H	5.96872600	-0.66644100	2.91913200
H	2.94106800	-0.14658500	5.03272900
C	3.51349500	-2.68658700	3.90513700
H	2.63846700	-3.32536300	4.11762200
H	4.20107500	-3.27013200	3.26689800
H	4.01757900	-2.48458900	4.86501300

48 atoms, **P_{2e} [CH_3, CH_3]**

PBE/6-31+G(d). E = -4729.168755 H

Mg	-3.65469500	-1.04214000	2.40894500

Mg	-3.34250400	-1.94616700	-0.14960300
Mg	-3.79261300	0.82142900	0.40989300
Mg	-1.27977600	-0.41429500	1.23088500
O	-2.40444900	-0.20858300	-0.66446500
O	-2.73718800	0.68959800	2.26660700
O	-2.25644000	-2.21610700	1.67903200
O	-4.74942000	-0.89617900	0.81394200
Mg	-0.86536900	-3.18920900	0.60599400
Mg	-0.45895500	-3.91390500	-1.99653200
Mg	-0.97122300	-1.25296300	-1.63347200
Mg	1.51892600	-2.53230000	-0.79534500
O	0.49737100	-2.32127500	-2.65008000
O	0.15289900	-1.46908100	0.30097800
O	0.55286600	-4.22527700	-0.36312500
O	-2.01528000	-3.03086000	-1.17787800
Mg	-1.76860900	2.36650300	1.74766600
Mg	-1.40746200	1.51428500	-1.05848400
Mg	-1.73115100	4.12306500	-0.33478800
Mg	0.65027000	3.03357500	0.39812300
O	-0.35858300	3.27906500	-1.46548300
O	-0.77973900	4.06375000	1.35956000
O	-0.29108700	1.28910800	0.87193800
O	-2.91274100	2.57058700	-0.04007700
Mg	1.08860500	0.25411700	-0.18528100
Mg	1.55877300	-0.57752200	-2.89277800
Mg	1.14875800	2.17741500	-2.29243300
Mg	3.45414200	0.82920200	-1.34516900
O	2.60231300	1.11272100	-3.14587600
O	2.12836500	2.03062800	-0.44854400
O	2.54987200	-0.91204200	-1.11579900
O	0.00641700	0.47299600	-2.04984000
C	4.06033500	0.10879300	1.66047900
O	4.57475300	0.40259100	0.56587900
C	3.25937200	-0.15825600	3.58918400
C	2.99590100	-0.87677800	2.22974600
H	2.43371600	0.47711900	3.95845300
H	1.97016300	-0.74522100	1.84219900
N	4.31101200	0.61039300	2.90408800
C	5.28197900	1.55963600	3.39564600
H	4.78757200	2.45239900	3.82136400
H	5.91410600	1.09566300	4.17408100
H	5.91644700	1.86805900	2.55048400
H	3.63621700	-0.80426500	4.40220800
C	3.37396200	-2.35774900	2.20736700
H	2.61734400	-2.95954700	2.73914000
H	3.44758000	-2.73088200	1.17196900
H	4.35477800	-2.52686500	2.68759700

48 atoms, **TS_{2e} [CH_3, CH_3]**

PBE/6-31+G(d). E = -4729.04781 H

Mg	-3.57886400	-1.04095200	2.43185700
Mg	-3.27161700	-1.95820500	-0.11010000
Mg	-3.75561000	0.80275200	0.43562100
Mg	-1.19971600	-0.38449200	1.24023800
O	-2.33909000	-0.22326100	-0.60890400
O	-2.67712100	0.69625300	2.28614400

O	-2.16828000	-2.20376400	1.70641300
O	-4.69366900	-0.92220300	0.84644000
Mg	-0.75754600	-3.14673400	0.63491700
Mg	-0.38027500	-3.89677100	-1.96397300
Mg	-0.92091100	-1.22491600	-1.63260600
Mg	1.60380300	-2.45866600	-0.82174100
O	0.53748200	-2.29945900	-2.63546500
O	0.31962600	-1.43409800	0.41699300
O	0.66266700	-4.17207400	-0.34275600
O	-1.92756500	-3.01699300	-1.13465600
Mg	-1.73648100	2.38734200	1.74128300
Mg	-1.37351800	1.50696500	-1.04249900
Mg	-1.78850500	4.12464600	-0.36177400
Mg	0.63469400	3.10903500	0.31587600
O	-0.43198000	3.29983400	-1.51099100
O	-0.80063000	4.10484900	1.31450300
O	-0.22334900	1.33528100	0.88493600
O	-2.91420500	2.54690200	-0.02502900
Mg	1.32100800	0.31893200	0.00288400
Mg	1.54289400	-0.52567100	-2.91806700
Mg	1.06497700	2.20023400	-2.36715100
Mg	3.43099200	0.97893500	-1.51464900
O	2.52860200	1.17894100	-3.27628800
O	2.10675900	2.17542000	-0.60111900
O	2.62414100	-0.83324700	-1.21208900
O	-0.01596600	0.48829300	-2.09631300
C	3.50399800	0.44063200	1.17732800
O	4.47093900	0.70364900	0.42632200
C	2.72324300	-0.04446700	3.44877600
C	2.63871700	-1.37264200	2.80489300
H	1.87905300	0.63689000	3.26724700
H	1.92705900	-1.49012400	1.96807500
N	3.87048100	0.23511200	2.56127100
C	5.21865500	0.44836000	3.11428400
H	5.27616400	1.41668100	3.64434700
H	5.46167500	-0.36003400	3.82403600
H	5.92868600	0.43908000	2.27497400
H	3.03947300	-0.03812400	4.50316200
C	3.42322500	-2.55030000	3.24719100
H	2.71072900	-3.27080800	3.70463800
H	3.87769500	-3.08782800	2.39574400
H	4.19462700	-2.32230100	4.00089700

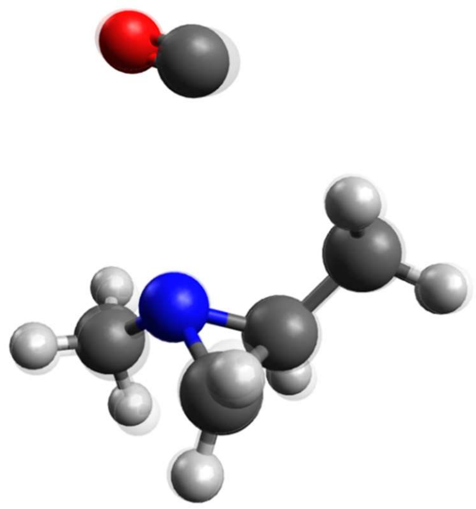

3-methylaziridine + CO

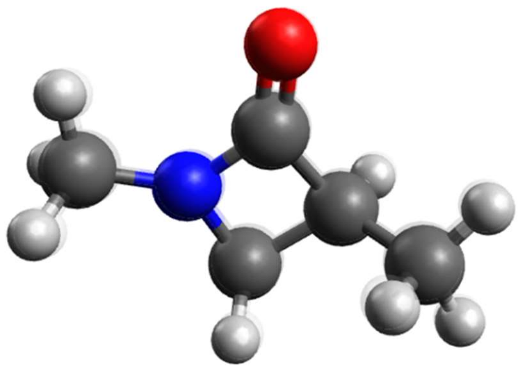

3-methyl-azetidi-2-one

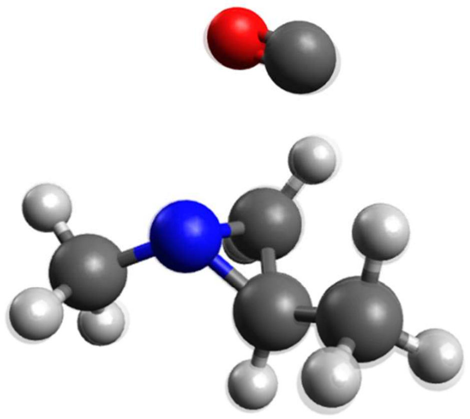

4-methylaziridine + CO

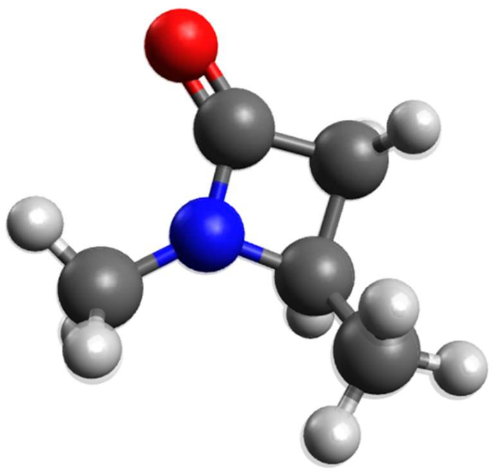

4-methyl-azetidi-2-one

Figure S1:

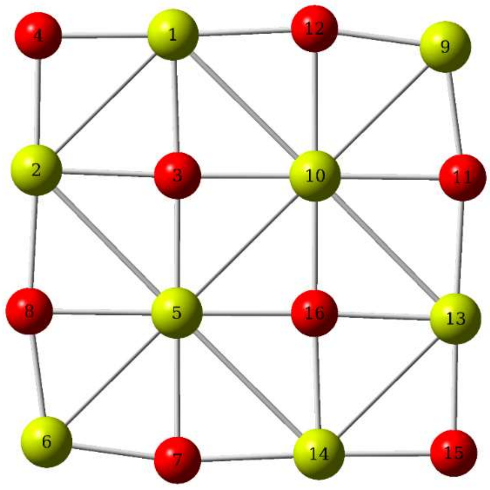

B3LYP/6-31G(d)

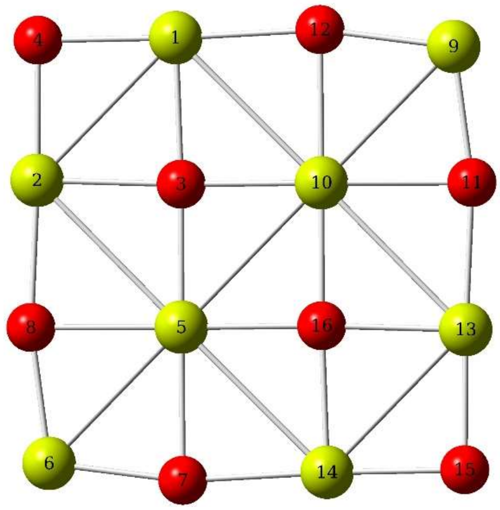

B3LYP/6-31+G(d)

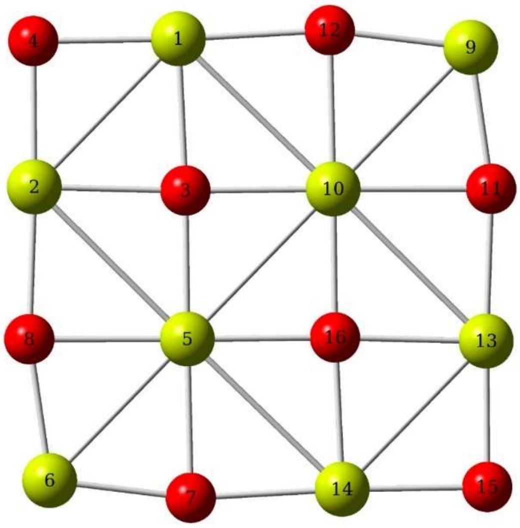

PBE/6-31+G(d)

Figure S2:

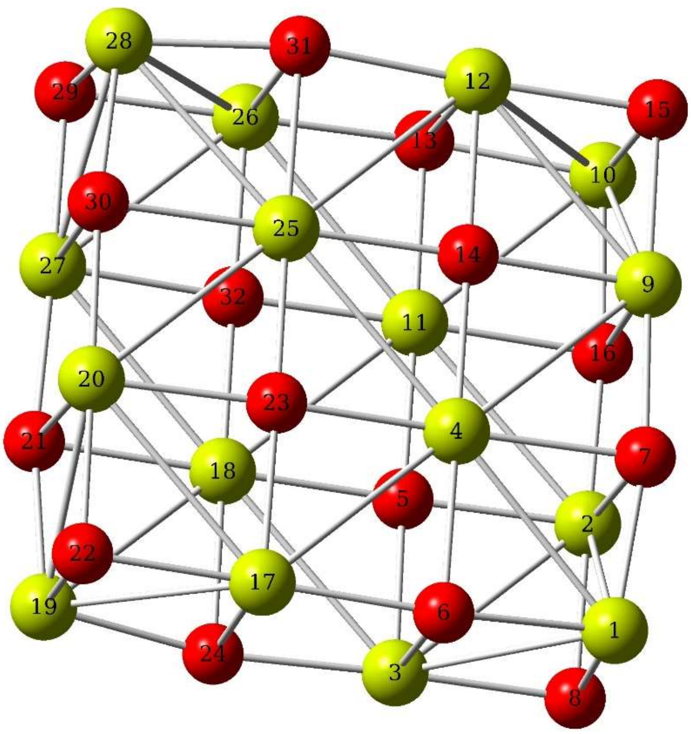
28
31
29
26
12
15
13
10
30
25
14
27
32
9
11
16
20
23
4
7
21
18
5
2
22
17
19
6
1
24
3
8

Figure S3:

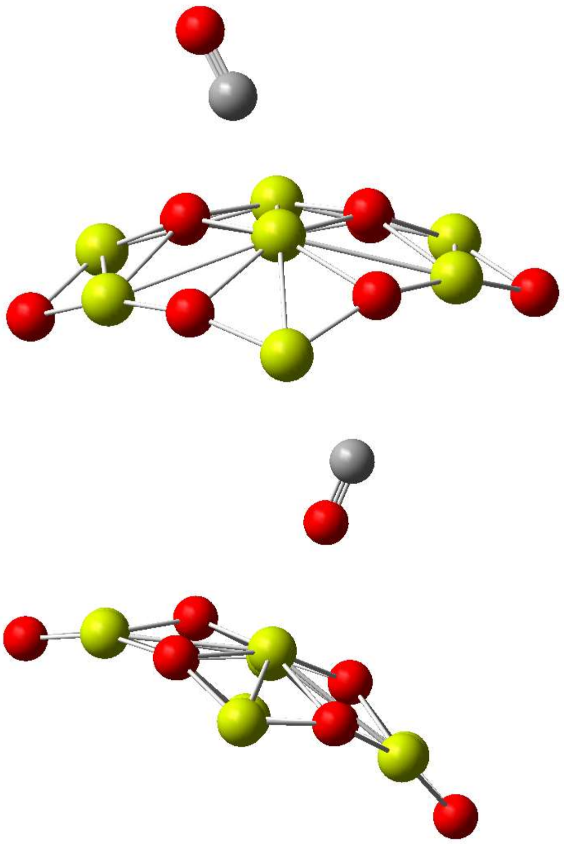

Figure S4:

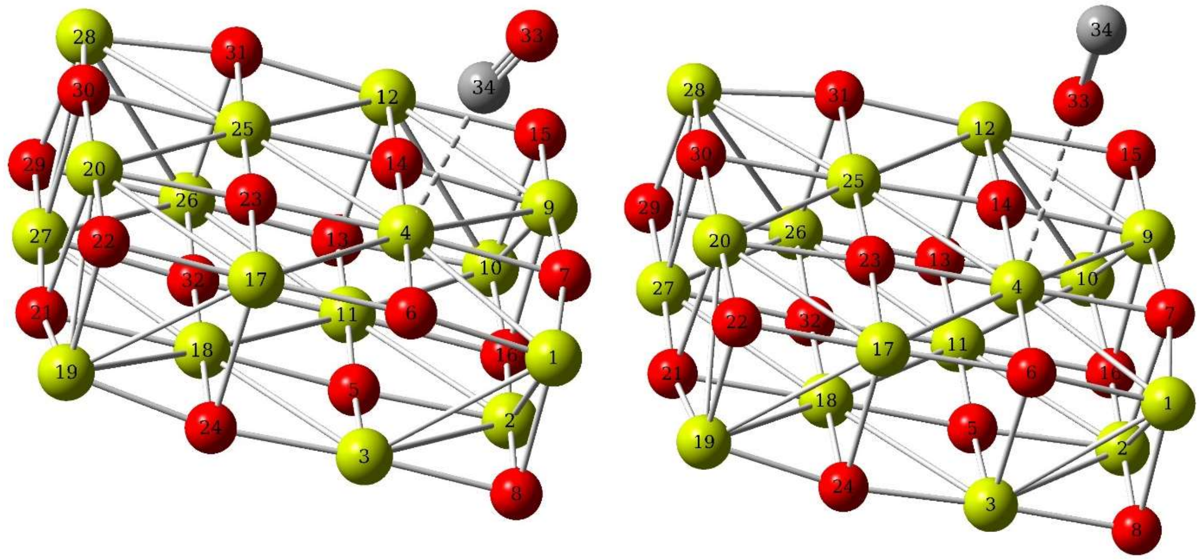

(*a*) (*b*)

Figure S5:

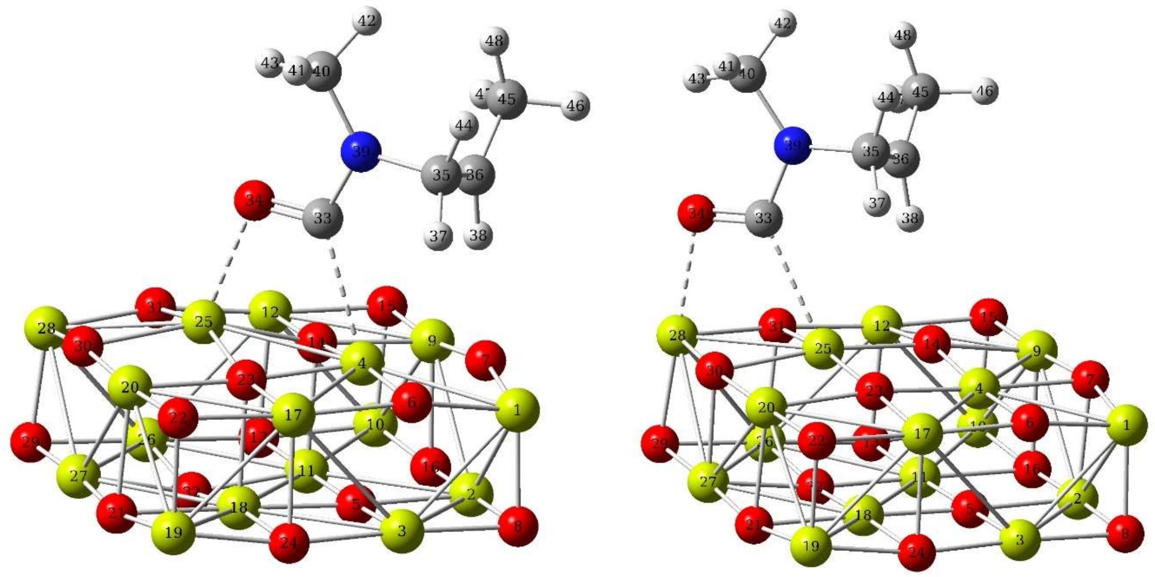

(*i*) (*ii*)

Figure S6:
